# Changes in palaeoclimate and palaeoenvironment in the Upper Yangtze area (South China) during the Ordovician–Silurian transition

**DOI:** 10.1038/s41598-022-17105-2

**Published:** 2022-08-01

**Authors:** Xin Men, Chuanlong Mou, Xiangying Ge

**Affiliations:** 1 College of Earth Science and Engineering, Shandong University of Science and Technology, 579 Qianwangang Road, Huangdao District, Qingdao, 266590 Shandong Province People’s Republic of China; 2 Chengdu Institute of Geology and Mineral Resources, China Geological Survey, Chengdu, 610081 Sichuan Province People’s Republic of China

**Keywords:** Climate sciences, Environmental sciences, Ocean sciences

## Abstract

The Ordovician–Silurian transition was a critical period in geological history, during which profound changes in climatic, biotic, and oceanic conditions occurred. To explore the provenance, palaeoclimate, and palaeoredox conditions in the Sichuan Basin during the Late Ordovician–early Silurian interval, we conducted mineralogical, geochemical, and isotopic analyses of three formations (Wufeng, Guanyinqiao and Longmaxi formations) in the Xindi No. 2 well. The ternary and bivariate diagrams indicate that the provenance is mainly felsic igneous rocks and originated mainly from a collisional setting, presumably due to an active continental margin. The chemical index of alteration (CIA) values in the lower Wufeng and Longmaxi formations are relatively high (67.48–73.57), indicating a warm and humid climate. In contrast, the CIA values declined rapidly (58.30–64.66) during the late Katian to early Hirnantian, which had a fluctuating cold and dry climate and was interrupted by a transient warm and humid climate. The palaeoredox indices (Mo concentrations and Mo_auth_/U_auth_, U/Th, V/Cr, Ni/Co, and V/V + Ni values) during the Late Ordovician–early Silurian indicate two cycles of water column euxinia. The first cycle occurred during Wufeng Formation deposition, with bottom waters evolving from oxic-suboxic to suboxic-anoxic. Most samples show relatively low redox-sensitive trace element concentrations during the Guanyinqiao Formation, pointing to oxic-suboxic conditions. The second cycle, during the late Hirnantian, transitioned from oxic to euxinic water conditions. Our δ^13^C_org_ data are comparable to previously reported records and exhibit a strong correlation between the Hirnantian isotopic carbon excursion (HICE), climate change, and redox conditions. We suggest that the variations in the δ^13^C values are related to two elements: (1) increased photosynthetic activity under oxic water conditions, and (2) increased carbonate weathering exposed by the glacio-eustatic sea- level. In addition, the high δ^13^C_org_ values might indicate a more shelf-proximal setting during Xindi No. 2 well deposition. The δ^13^C_org_ isotopic data effectively constrain the timing of the Late Ordovician mass extinction (LOME) and the evolution of the temporal changes in the climatic and ocean redox conditions, suggesting an apparent stratigraphic coincidence between climate and redox fluctuations and two-phase extinctions, which implies a strong causal relationship. The LOME was systematically driven by the combination of cooler glacial temperatures, glacio-eustatic sea-level fluctuations, and anoxic water conditions that caused the two pulses of extinction in the Yangtze shelf sea.

## Introduction

The Late Ordovician (Katian-Hirnantian stage) to early Silurian (early Rhuddanian) was an important interval in Earth history, during which the environment, biodiversity, and plate tectonics changed considerably^1–3^. The Late Ordovician mass extinction (LOME) was the first of the five considerable Phanerozoic extinction events, along with the Hirnantian glaciation event, major sea level changes, and the extensive deposition of organic-rich shales^2,3^. It comprised two discrete pulses: the first pulse started during or just before the *Metabolograptus extraordinarius* graptolite biozone^4–8^ and mainly affected benthic, nektonic and planktonic species^9,10^. The second pulse, initiating at the start of the *M. perculptus* biozone, is associated with the extinction of a distinctive fossil assemblage known as the Hirnantia Fauna, including diverse and abundant brachiopods and trilobites^11,12^. Hirnantia Fauna has been interpreted to be mainly cool-water benthic fauna that began to spread rapidly and widely after the first extinction event but suddenly became extinct during the second extinction event^6,8,11,12^. During this short time interval, approximately 85% of marine animal species and 25% of animal families went extinct^13,14^.

The past decade has witnessed research efforts toward understanding the context and nature of the LOME, and yet a consensus has not been reached on the causes of Earth's second most severe biotic catastrophe. A number of possible extinction mechanisms are associated with changes in the global climate and ocean chemistry. Numerous analyses have demonstrated that the Hirnantian glaciation was a relatively short but intense climatic event marked by an abrupt glacio-eustatic sea-level change, that was broadly coincident with the LOME^15^. These large fluctuations are recorded as major lithofacies changes in the South China Craton, where shelly limestones of the syn-glacial Guanyinqiao Formation are sandwiched between the graptolitic black shales of the pre-glacial Wufeng Formation and the post-glacial Longmaxi Formation and are known as deep-water graptolitic black shales^6,7,12^. Anoxia was widespread and expanded repeatedly in the Late Ordovician–early Silurian ocean during the deposition of black shales, and these events are commonly discussed as the preferential extinction of pelagic species, such as graptolites, as well as key benthic taxa, such as trilobites^16,17^. A stratified anoxic water column has also been confirmed based on geochemical evidence, such as high redox-sensitive trace element abundances (Mo, U, and V), heavy sulfur isotopic records (δ^34^Sδ), Fe speciation analysis, and distribution of pyrite framboid diameters, mainly from South China^17–20^. There is an apparent temporal correlation between large igneous province (LIP) eruptions and at least half of the major extinctions of the Phanerozoic. The LOME has not been correlated with a recorded LIP event, until recently, when Derakhshi^21^ reported a series of voluminous volcanic events of the Middle Ordovician–Silurian from northern Iran. These erosional and deformed remnants (volcanic rocks) distributed over a length of ca. 1700 km, identified as the newly proposed Alborz LIP, have a high potential to be the main cause of the LOME. Before the recorded LIP event had been discovered, many researchers postulated volcanism as the trigger for the LOME as a primary driver of global environmental and climate changes. Volcanism could lead to eutrophication, increased amounts of CO_2_ in the atmosphere, and as a consequence, increased anoxia^22–26^. However, the causes of the far-reaching catastrophic biological and environmental changes during the Late Ordovician to early Silurian remain controversial.

Carbon isotopes represent an important tool for correlating the Ordovician system, both at global^27,28^ and regional scales^29,30^. Previous studies have revealed the Hirnantian isotope carbon excursion (HICE), which is characterized by significant δ^13^C-enrichments of both sedimentary carbonates (δ^13^C_carb_) and organic carbon (δ^13^C_org_) that is broadly recorded in Ordovician–Silurian sections (e.g., Refs.^31–36^). The HICE was associated with a mass extinction, extensive glaciation of Gondwana, and a global sea-level low stand (e.g., Refs.^37–39^). The positive δ^13^C excursions have been interpreted to reflect the enhanced burial of organic carbon driven by expanded anoxic conditions^18,29^. However, both shallow and deep water columns have indicated increasing oxygenation during the Hirnantian glaciation based on redox-sensitive elements, iron speciation, nitrogen, molybdenum, and uranium isotope data (e.g., Refs.^40–43^). Therefore, the possible origin of the positive δ^13^C excursions and its potential relationships to environmental changes during the Hirnantian Stage require further investigation.

The temporal relationship between glaciation, marine anoxia, and the δ^13^C curve is clearly a subject of debate, as is the relationship between palaeoclimate, redox changes, and the O–S biotic crisis. We examine these topics here based on the Xindi No. 2 drill core section in the Sichuan Basin and aim to show the relationship between palaeoclimate, anoxia, and δ^13^C trends. A combined graptolite biostratigraphic and carbon isotope study is used to generate a framework. This framework, combined with mineralogy and diverse elemental data, provide new insights into the provenance, palaeoclimate, redox conditions, and palaeoenvironmental change during the Late Ordovician crisis interval.

## Geological setting

**Figure 1:**
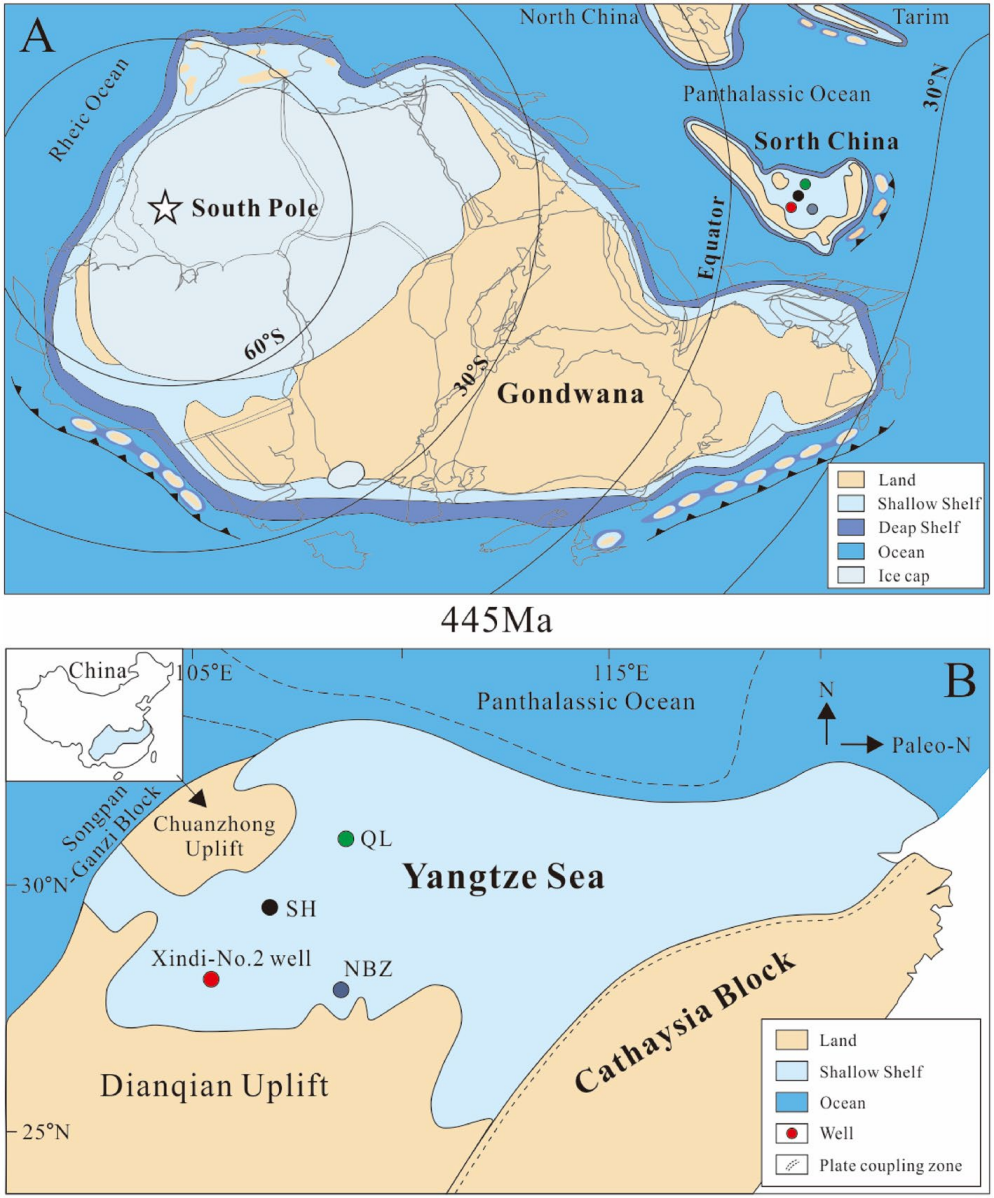
Geological setting. (**A**) Late Ordovician (445 Ma) palaeogeography with the location of South China (adapted from Torsvik and Cocks^45^). Red circle represents the Xindi-No. 2 well. (**B**) Simplified palaeogeographic map of the Yangtze Shelf Sea during the Ordovician–Silurian transition, showing the well localities.

The South China Block was an isolated microcontinent located off the northwestern margin of Gondwana near the equator in the Late Ordovician^44,45^. At that time, the South China Block comprised two subblocks, the Yangtze Block in the northwest and the Cathaysia Block in the southeast. The Yangtze Block formed a shallow carbonate platform during the Cambrian period^46–49^. During the Late Ordovician Kwangsian Orogeny, the Cathaysia Block collided with the Yangtze Block, resulting in a compression effect in the northwest-southeast orientation, which caused a relative rise in sea level and a partial uplift of the craton edge (e.g., Chuanzhong, Xuefeng, and Qianzhong uplifts; Fig. 1). As a result, the Yangtze carbonate platform evolved into a siliceous clastic shelf basin, and the Yangtze Sea was largely isolated from the open sea, forming an anoxic, stagnant, low-energy seawater environment, which eventually led to the deposition of thick and rich organic shales in this area^50–53^.

**Figure 2:**
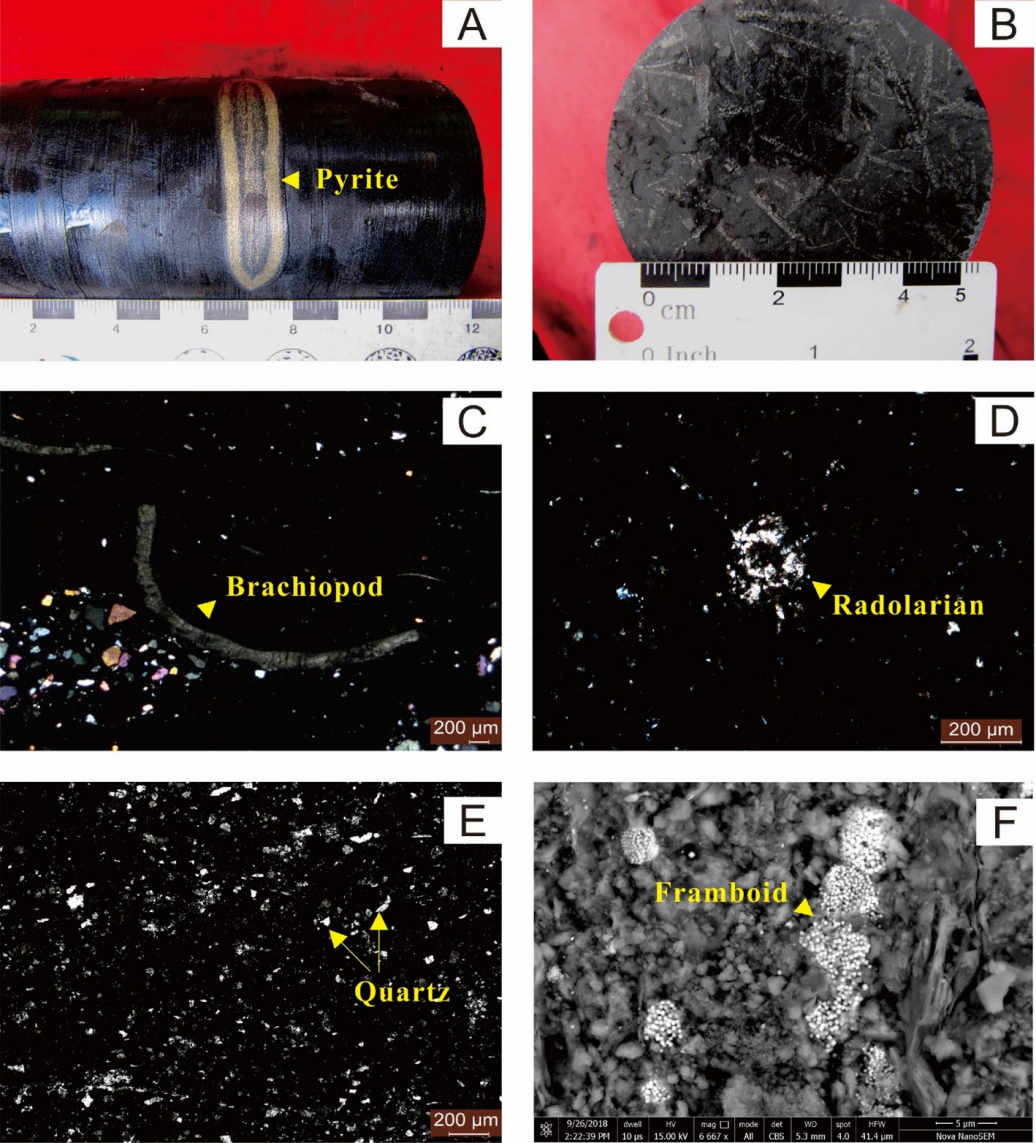
Sedimentary features of the Wufeng-Longmaxi formations in Xindi No. 2 well, Sichuan Basin, South China. (**A**) Pyrite occurs as girdles in the black shales of the Longmaxi Formation (**B**) Graptolites developed in the Wufeng Formation (**C**) A fragile piece of brachiopods (yellow arrow) in sample XD2P-B30. (**D**) Radiolarians developed in black shales of the Wufeng Formation (sample XD2P-B21). (**E**) Detrital quartz, moderately sorted and moderately rounded, is present in the black shales of the Longmaxi Formation (sample XD2P-B34). (**F**) SEM image of framboidal pyrite clusters.

The Xindi No. 2 well is located in Daguan District, Zhaotong City, Yunnan Province, in the southwestern Sichuan Basin. The strata across the Ordovician–Silurian boundary of the Xindi No. 2 well are the Wufeng, Guanyinqiao, and Longmaxi formations, in ascending order. The Wufeng Formation is mainly composed of dark-grey, carbonaceous shales with interlayers of siliceous shales and mudstone, containing abundant and diverse graptolite faunas and radiolarians (Fig. 2A,B,D). The overlying Guanyinqiao Formation is composed of carbonaceous marls, which contain cold-water adapted Hirnantia Fauna^6^ (Fig. 2C). The Longmaxi Formation, generally much thicker than the other two units, is subdivided into two parts. The lower part is composed of black, graptolite-rich, carbonaceous shales, siliceous shales, and carbonaceous mudstone. The upper part is composed of grey, green mudstone with fewer fossils. K-bentonite beds have been observed in the Wufeng Formation(nine beds) and in the lower part of the Longmaxi Formation (seven beds).

## Samples and methods

**Figure 3:**
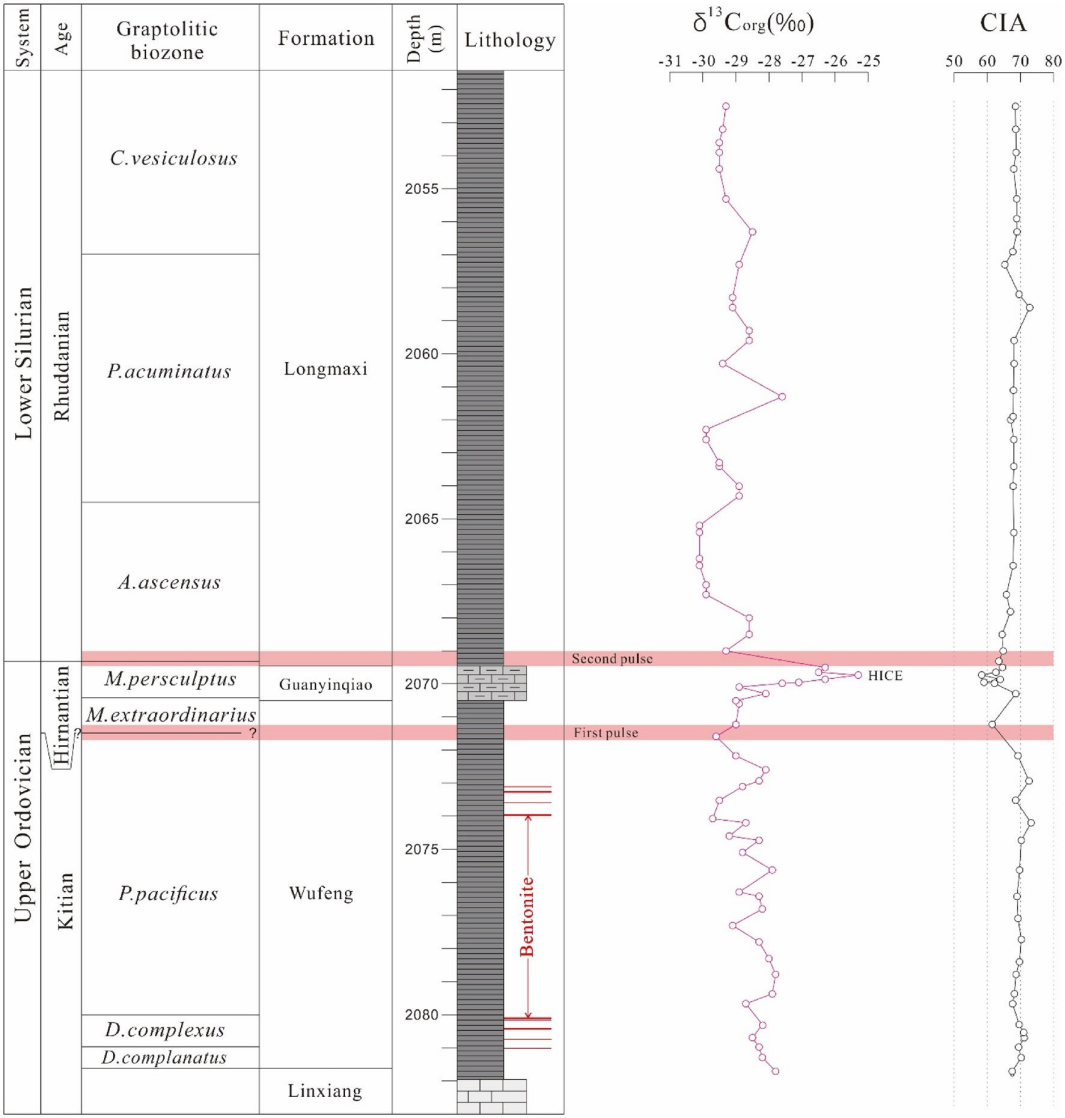
Biostratigraphic data, δ^13^C_org_ profiles, and CIA values for the Xindi No. 2 well. The biostratigraphy is based on Zhang et al.^54^. *HICE* Hirnantian isotopic curve excursion.

A total of 54 samples were collected from the Xindi No. 2 well. Twenty-one samples were obtained from black shales of the Wufeng Formation with an average interval of 0.5 m, seven carbonaceous marls from Guanyinqiao Formation were collected with an average interval of 0.1 m and 26 black shales of the Longmaxi Formation were taken with an average interval of 0.6 m. The above sampling location is given in Fig. 3. Samples were coarsely crushed through a steel jaw crusher and then powdered in an agate mill down to a grain size smaller than 200 mesh for geochemical analysis.

Fifty-four samples of major oxides including Al_2_O_3_, CaO, Fe_2_O_3_, K_2_O, MgO, MnO, Na_2_O, P_2_O_5_, SiO_2_ and TiO_2_ were measured by a PW4400 X-ray fluorescence spectrometer at the National Research Center of Geoanalysis, China. The sample preparation for major elements was conducted using Chinese National Standard GB/T 14506.28-2010. The analytical uncertainty was generally less than 5%. A total of 54 samples of trace element compositions (including rare earth elements [REEs]) were measured using a PE300D inductively coupled plasma mass spectrometer, strictly followed by Chinese National standard GB/T 14506.30-2010.

A Nova Nano SEM450 scanning electron microscope (15 kV and 10^–10^ A) was applied to study the morphology and distribution of typical minerals in ten selected samples in the laboratory of the Beijing Institute of Uranium Geology. The analytical procedures were followed by the Chinese National standard GB/T 18295-2001 and GB/T 17359-2013. The mineral compositions of the whole-rock and clay minerals of seventeen samples were determined using the ZJ207 Bruker D8 advance automated powder diffractometer equipped with Ni-filtered Cu–Ka radiation (40 kV accelerating voltage and 40 mA beam current) at Chongqing Mineral Resources Supervision and Test Center, China. The X-ray diffraction (XRD) pattern was scanned from 3° to 45° with a step size of 0.02° (2θ), and the humidity was controlled at 39% RH during the determination of the XRD pattern.

Fifty-four samples of stable organic carbon isotope measurements were performed in the laboratory of the Beijing Institute of Uranium Geology. Approximately 200 mg of the samples were placed in a container containing ~ 250 ml 1 N HCl solution to remove the carbonate minerals. After standing for 48 h, the material was filtered through a vacuum pump and rinsed with deionized water to remove excess acid and chlorine. The remaining precipitate was dried, separated from the filter, and stored in 20 ml glass vials. The δ^13^C values of the organics separated from the decarbonated samples were analyse on a Thermo Fisher MAT-253 mass spectrometer. The Chinese national standard GB/T 18340.2-2010 was used to monitor analytical accuracy. Analytical precision was better than ± 0.1‰ for δ^13^C_org_ based on replicate analyses of standards and samples.

The graptolites from the Xindi No. 2 well were collected and identified by Zhang et al.^54^ for each sample. Nine graptolite biozones are recognized in the Xindi No. 2 well, including *Dicellograptus, complanatus, D. complexus, P. pacificus*–*M. extraordinarius, A. ascensus*–*P. acuminatus, C. vesiculosus, C. cyphus, D. triangulatus, L. convolutes, and S. sedgwickii* biozones. However, *Metabolograptus extraordinarius* (Sobolevskaya) and *Metabolograptus persculptus* (Salter) have not been found. The appearance of *Normalograptus mirnyensis* (Obut and Sobtolevskaya) at the depth of 2070.73 m confirmed that the *M. perculptus* biozone has been reached. The graptolite biozonation, thus, could be correlated with other Wufeng, Guanyinqiao, and Longmaxi Formation data.

## Results

### Mineralogy

**Figure 4:**
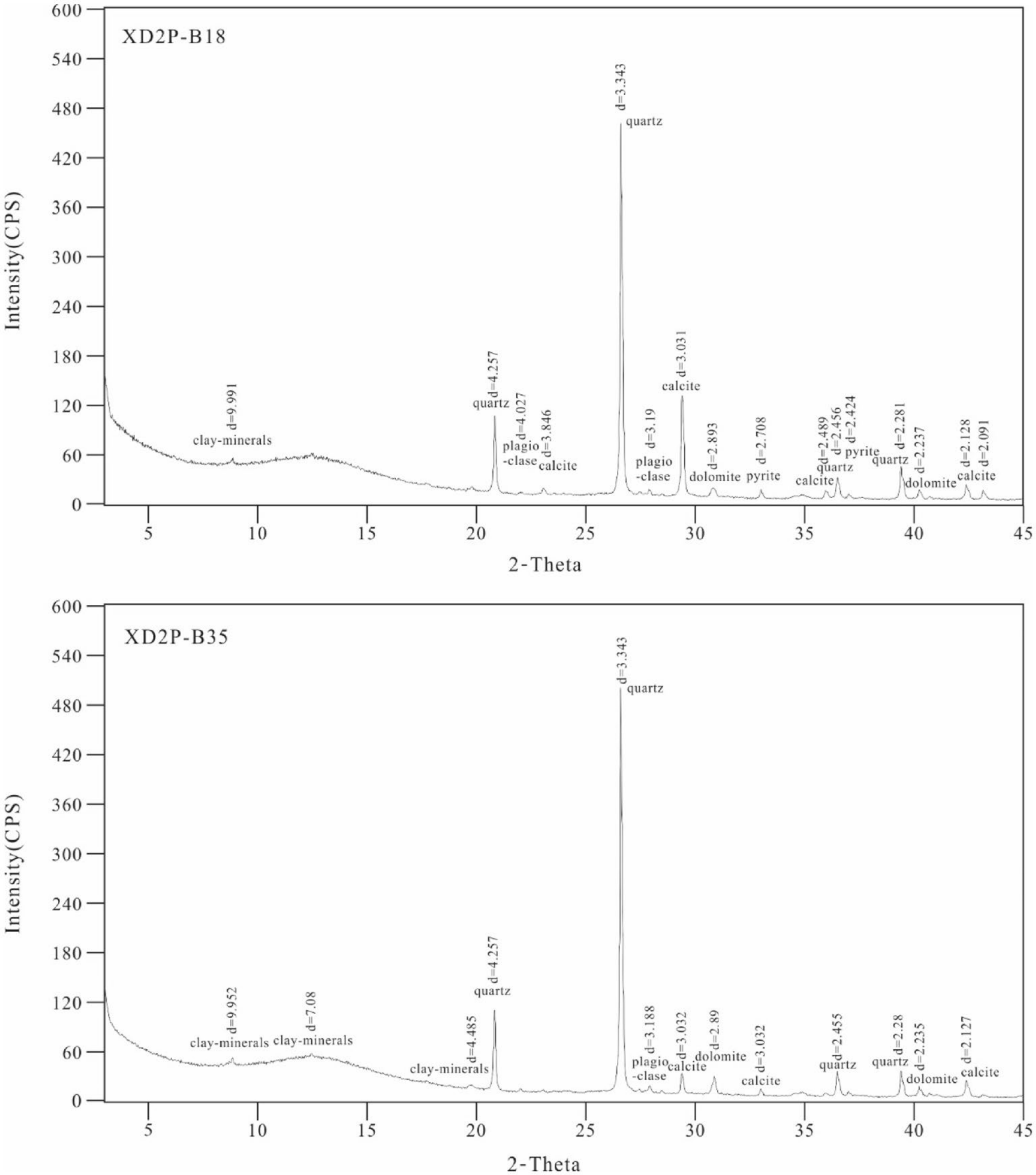
XRD patterns of studied samples XD2P-B18 and XD2P-B35 (the marks in the figure are eigenvalues and corresponding minerals).

The XRD analysis results are illustrated in Table 1 and Fig. 4. No significant qualitative difference was found in the mineralogical composition of samples from the Wufeng- Longmaxi formations. The samples are composed mainly of quartz (Fig. 2E, 22.1–71.3%, average of 39.1%), clay minerals (11.2–38.4%, average of 20.6%), calcite (4–28%, average of 17.7%) and dolomite (2.3–27.2%, average of 14.3%). Moreover, minor albite (1.6–6%, average of 3.6%), pyrite (0.5–5.9%, average of 3.3%) and microcline (0.4–3.4%, average of 1.3%) occur in the samples. The mixed layers of illite/smectite (43.0–65.0% average of 53.6%) and illite (22–40%, average of 30.8%) are the dominant constituents of the clay mineral contents of the Wufeng-Longmaxi formations. In addition, small numbers of minerals, including pyrite, molybdenite, and ilmenite, were detected simultaneously by XRD and scanning electron microscopy (SEM). Pyrite is mainly present as typical framboids and single euhedral pyrites in the studied samples (Fig. 2F).

**Table 1:** Mineral compositions of the shales and carbonaceous marls from the Wufeng-Longmaxi formations in the Xindi No. 2 well (XRD).

Sample	Depth	Quantitative analysis of the whole rock (%)								Relative content of clay minerals (%)						%S	
		Quartz	Microline	Albite	Calcite	Dolomite	Pyrite	Pyroxene	Clay	K	C	I	S	I/S	C/S	I/S	C/S
XD2-B60	2052.63	29.1	1.5	6.0	11.1	13.0	5.8	–	33.5	1	10.0	23.0	–	57.0	9.0	6	9.0
XD2-B58	2053.63	35.6	1.1	4.5	10.5	10.1	4.7	–	33.5	2	9.0	26.0	–	49.0	14.0	5	10.0
XD2-B57	2054.42	37.8	0.9	3.5	12.8	12.8	5.9	–	26.3	1	6.0	26	–	58.0	9	6	19.0
XD2-B51	2058.3	37.2	0.6	2.1	28.0	17.0	2.3	–	12.8	2	4.0	39	–	43.0	12	6	14.0
XD2-B49	2059.28	29.8	0.4	2.8	23.0	27.2	3.2	–	13.6	2	5.0	32.0	–	54.0	7.0	6	11.0
XD2-B40	2065.21	38.0	1.0	4.1	24.3	17.0	2.3	–	13.3	2	8.0	31.0	–	53.0	6.0	5	14.0
XD2-B39	2066.21	46.0	0.8	5.2	10.5	11.6	3.7	–	22.2	1	2.0	34	–	56.0	7	6	14.0
XD2-B38	2067.04	41.7	0.9	3.1	23.9	14.8	4.4	–	11.2	2	4.0	30.0	–	60.0	4.0	5	9.0
XD2-B37	2068.04	40.8	0.8	2.6	27.3	8.8	3.1	–	16.6	2	4.0	30.0	–	52.0	12.0	5	9.0
XD2-B35	2069.02	65.2	1.3	3.2	4.2	7.6	3.7	–	14.8	2	7.0	32.0	–	49.0	10.0	5	11.0
XD2-B27	2070.49	34.1	3.4	4.7	18.1	17.1	3.0	–	19.6	1	3.0	26	–	63.0	7	6	9.0
XD2-B23	2073.58	43.9	1.5	1.9	20.0	13.1	3.1	–	16.5	3	8.0	40.0	–	44.0	5.0	5	12.0
XD2-B19	2076.35	54.4	0.7	1.6	8.9	14.2	2.2	–	18.0	2	4.0	22.0	–	65.0	7.0	5	11.0
XD2-B18	2077.3	53.4	1.0	2.8	20.2	4.7	2.9	–	15.0	2	7.0	35.0	–	44.0	12.0	5	16.0
XD2-B13	2079.6	71.3	1.3	3.4	4.0	2.3	1.4	–	16.3	1	4.0	22	–	65.0	8	5	17.0
XD2-B8	2080.74	27.3	1.5	3.5	20.5	6.6	1.8	3.5	35.3	1	5.0	32.0	–	57.0	5.0	6	12.0
XD2-B4	2081.76	23.0	1.9	5.9	19.4	10.9	0.5	–	38.4	–	5.0	28.0	–	60.0	7.0	7	9.0

*K* kaolinite, *C* clinochlore, *I* illite, *S* smectite, *I/S* illite/smectite mixed layer, *C/S* clinochlore/smectite mixed layer, *%S* mixed-layer ratio.

### Geochemistry

#### Major elements

The major element concentrations of samples from the Xindi No. 2 well are listed in Table 2. For the Wufeng and Longmaxi formations, SiO_2_ (20.79–81.98% and 39.9–75.11%, respectively), CaO (1.85–33.58% and 4.48–21.8%, respectively) and Al_2_O_3_ (1.83–14.6% and 3.39–20.55%, respectively) are the most abundant oxides.

**Table 2:** Analytical data of major elements (wt%) and organic carbon isotopes (‰) in shales and carbonaceous marls from the Wufeng-Longmaxi formations in the Xindi No. 2 well.

Sample	Height(m)	SiO_2_	SiO_2-adj_	Al_2_O_3_	CaO	Fe_2_O_3_	FeO	K_2_O	MgO	MnO	Na_2_O	P_2_O_5_	TiO_2_	LOI	Al_2_O_3_/TiO_2_	CaO*	CIA	δ^13^C_org_
XD2P-B60	2052.48	53.24	63.12	13.05	5.73	2.35	1.9	3.59	3	0.03	0.64	0.08	0.53	13.31	24.62	0.010	68.50	− 29.3
XD2P-B59	2053.22	54.45	64.34	11.6	6.82	2.51	1.58	3.23	3.09	0.03	0.55	0.09	0.5	13.98	23.20	0.009	68.58	− 29.3
XD2P-B58	2053.92	55.05	65.07	11.51	7.28	2.11	1.33	3.18	2.92	0.03	0.54	0.07	0.44	13.96	26.16	0.009	68.77	− 29.5
XD2P-B57	2054.42	54.56	64.85	9.77	9.5	2.45	1.47	2.75	2.47	0.03	0.49	0.06	0.42	14.36	23.26	0.008	68.01	− 29.5
XD2P-B56	2055.32	41.93	54.24	8.7	14.61	1.53	1.62	2.52	5.31	0.07	0.36	0.08	0.4	21.22	21.75	0.006	68.94	− 29.2
XD2P-B55	2055.92	42.2	54.50	8.57	14.68	1.66	1.54	2.44	5.28	0.06	0.37	0.07	0.39	21.1	21.97	0.006	68.92	–
XD2P-B54	2056.32	40	52.44	7.66	17.53	2.01	1.11	2.2	4.86	0.06	0.32	0.06	0.34	22.34	22.53	0.005	69.01	− 28.5
XD2P-B53	2056.92	41.29	54.34	6.96	17.08	1.55	1.11	1.96	5.1	0.06	0.36	0.07	0.32	22.71	21.75	0.006	67.76	–
XD2P-B52	2057.32	55.53	67.22	7.94	10.27	1.75	0.93	2.24	2.84	0.03	0.54	0.07	0.36	16.08	22.06	0.009	65.36	− 28.9
XD2P-B51	2058.22	42.71	55.86	5.97	18.94	0.8	1.29	1.64	4.35	0.07	0.25	0.05	0.25	22.36	23.88	0.004	69.64	− 29.1
XD2P-B50	2058.62	43.03	49.70	20.55	4.67	8.05	0.9	5.36	2.91	0.08	0.56	0.03	0.34	11.69	60.44	0.009	72.85	− 29.1
XD2P-B49	2059.58	41.35	54.72	5.49	19.31	1.53	0.65	1.58	4.97	0.06	0.26	0.05	0.25	23.02	21.96	0.004	68.11	− 28.6
XD2P-B48	2060.32	43.55	56.32	6.47	17.92	1.52	1.04	1.85	4.14	0.06	0.31	0.05	0.3	21.2	21.57	0.005	68.12	− 29.4
XD2P-B47	2061.12	41.49	55.14	5.44	18.85	1.04	0.93	1.58	5.19	0.05	0.26	0.05	0.26	23.36	20.92	0.004	67.92	− 27.6
XD2P-B46	2061.92	41.14	54.46	5.12	21.01	0.87	1.04	1.51	4.16	0.05	0.24	0.05	0.24	23.52	21.33	0.004	67.83	–
XD2P-B45	2062.02	44.32	58.14	4.92	18	0.8	1.26	1.43	4.77	0.05	0.26	0.05	0.23	22.41	21.39	0.004	67.15	–
XD2P-B44	2062.60	42.42	55.71	6.24	17.27	1.06	0.97	1.82	5.57	0.05	0.29	0.05	0.3	22.98	20.80	0.005	68.05	− 29.9
XD2P-B43	2063.42	40.02	53.52	5.21	19.86	0.54	1.26	1.53	5.62	0.06	0.24	0.05	0.25	24.3	20.84	0.004	68.02	− 29.5
XD2P-B42	2064.07	40.21	53.56	4.89	21.13	0.9	0.9	1.43	4.93	0.06	0.23	0.05	0.24	24.13	20.38	0.004	67.93	− 28.9
XD2P-B40	2064.92	39.9	53.21	5.43	21.8	0.98	0.83	1.62	3.73	0.06	0.24	0.05	0.25	23.84	21.72	0.004	68.07	− 30.1
XD2P-B39	2065.42	46.16	59.70	6.18	17.26	0.78	1.11	1.82	3.21	0.05	0.29	0.05	0.29	22.07	21.31	0.005	67.84	− 30.1
XD2P-B38	2066.42	72.62	82.75	4.95	5.46	0.77	0.57	1.43	1.28	0.02	0.31	0.05	0.23	11.14	21.52	0.005	65.81	− 29.9
XD2P-B37	2067.82	69.1	80.34	3.47	9.87	0.05	0.86	1	1.13	0.03	0.19	0.05	0.16	13.28	21.69	0.003	66.98	− 28.6
XD2P-B36	2068.52	75.11	84.83	4.57	4.48	0.53	0.68	1.31	1.22	0.02	0.3	0.05	0.2	10.84	22.85	0.005	65.49	− 28.6
XD2P-B35	2069.02	68.41	79.39	5.98	5.53	0.87	1.04	1.74	1.68	0.02	0.41	0.1	0.27	13.18	22.15	0.007	64.88	− 29.3
XD2P-B34	2069.37	44.4	59.89	3.39	20.11	0.57	0.9	1.04	3.06	0.06	0.25	0.1	0.16	25.33	21.19	0.004	63.47	− 29.5
XD2P-B33	2069.52	50.43	62.13	6.88	16.74	0.89	0.83	2.01	2.31	0.06	0.48	0.1	0.35	17.69	19.66	0.008	64.66	− 26.3
XD2P-B32	2069.67	44.73	56.84	6.15	18.26	3.09	0.9	1.81	2.61	0.07	0.52	0.14	0.32	18.7	19.22	0.008	62.60	− 26.5
XD2P-B31	2069.77	57.88	65.01	8.88	2.28	12.96	0.86	2.61	0.91	0.06	1.07	0.94	0.49	9.53	18.12	0.017	58.30	− 25.3
XD2P-B30	2069.87	45.56	57.63	6.26	18.64	1.85	1.26	1.86	2.56	0.07	0.46	0.1	0.3	19.85	20.87	0.007	63.93	− 26.3
XD2P-B29	2069.94	58.11	66.25	9.36	4.94	8.38	0.83	2.74	1.33	0.06	1.07	0.3	0.5	10.32	18.72	0.017	59.04	− 27.1
XD2P-B28	2069.98	36.66	49.98	7.68	18.54	2.42	1.11	2.09	3.38	0.06	0.73	0.15	0.4	24.46	19.20	0.012	62.19	− 27.6
XD2P-B27	2070.32	44.14	53.97	14.39	12.25	0.85	1.11	4.12	3.7	0.03	0.63	0.09	0.35	17.4	41.11	0.010	68.74	− 28.1
XD2P-B26	2071.22	20.79	32.47	1.83	33.58	0.85	0.47	0.54	5.5	0.1	0.17	0.04	0.1	35.04	18.30	0.003	61.51	− 29
XD2P-B25	2072.17	37.44	51.54	3.77	26.18	0.7	0.75	1.12	2.12	0.11	0.14	0.05	0.18	24.81	20.94	0.002	69.23	− 29
XD2P-B24	2072.92	51.57	63.81	6.05	17.55	0.66	0.75	1.46	2.15	0.07	0.21	0.05	0.22	18.66	27.50	0.003	72.67	− 28.3
XD2P-B23	2073.55	62.4	72.56	7.13	8.64	1.24	1.11	2.13	2.46	0.05	0.31	0.06	0.34	13.08	20.97	0.005	68.16	− 29.5
XD2P-B22	2074.20	51.53	63.43	6.57	15.44	0.89	0.97	1.53	3.59	0.09	0.22	0.05	0.25	18.36	26.28	0.004	73.37	− 28.7
XD2P-B21	2074.77	45.32	58.18	5.33	19.49	0.93	0.68	1.58	3.9	0.11	0.16	0.04	0.28	21.33	19.04	0.003	70.40	− 28.3
XD2P-B20	2075.62	36.01	47.00	7.1	21.78	4.54	0.83	2.11	3.12	0.13	0.24	0.14	0.52	21.41	13.65	0.004	69.75	− 27.9
XD2P-B19	2076.42	67.52	77.20	5.57	7.93	0.98	0.97	1.66	2.15	0.06	0.21	0.05	0.25	11.58	22.28	0.003	69.09	− 28.3
XD2P-B18	2077.1	71.53	80.26	6.05	5.43	0.98	0.97	1.78	1.66	0.04	0.23	0.07	0.28	10.16	21.61	0.004	69.24	− 29.1
XD2P-B17	2077.7	40.39	51.88	8.69	18.32	2.08	0.9	2.58	4.01	0.11	0.26	0.06	0.36	20.91	24.14	0.004	70.39	− 28.2
XD2P-B16	2078.4	42.19	53.58	8.75	17.55	1.12	1.26	2.62	4.22	0.16	0.29	0.04	0.4	20.08	21.88	0.005	69.74	− 28
XD2P-B15	2078.79	57.67	67.74	8.97	9.42	0.08	2.05	2.73	3.08	0.07	0.34	0.07	0.43	14.05	20.86	0.005	68.73	− 27.8
XD2P-B14	2079.38	66.66	75.25	7.67	7.89	0.25	1.11	2.37	1.78	0.06	0.31	0.04	0.32	10.49	23.97	0.005	68.11	− 27.9
XD2P-B13	2079.67	81.98	87.15	5.78	1.85	0.06	1.04	1.7	0.93	0.02	0.24	0.04	0.31	5.11	18.65	0.004	68.69	− 28.7
XD2P-B12	2080.3	56.31	63.29	14.03	6.97	1.14	1.76	4.15	3.31	0.05	0.48	0.05	0.53	10.91	26.47	0.008	69.76	− 28.2
XD2P-B10	2080.57	42.59	51.83	12.86	14.87	0.54	2.34	3.58	3.53	0.11	0.41	0.65	0.43	16.27	29.91	0.007	71.07	− 28.3
XD2P-B8	2080.7	38.44	48.81	10.33	19.86	0.7	2.05	2.93	3.3	0.14	0.31	0.04	0.42	19.67	24.60	0.005	71.10	− 28.5
XD2P-B6	2080.99	42.46	52.29	11.52	13.84	1.51	2.05	3.29	5.17	0.12	0.44	0.05	0.52	17.7	22.15	0.007	69.66	− 28.3
XD2P-B5	2081.32	35.58	45.10	10.52	18.16	2	2.12	2.87	6.2	0.26	0.4	0.03	0.52	20.49	20.23	0.006	70.37	− 28.2
XD2P-B4	2081.77	43.63	52.27	13.55	13.84	0.97	1.94	3.98	3.89	0.09	0.67	0.05	0.65	15.31	20.85	0.011	67.50	− 27.8
XD2P-B3	2081.82	47.12	54.96	14.6	11.01	0.86	2.23	4.27	3.81	0.07	0.73	0.05	0.73	13.56	20.00	0.012	67.48	− 28.2

According to XRD analysis data, the mineral compositions of shales from the Wufeng and Longmaxi formations are mainly composed of quartz, clays, calcite, and dolomite, which is consistent with the major element compositions (Table 2). Based on the XRD analysis data, the mineral compositions of shales from the Wufeng and Longmaxi formations are dominated by quartz, clays, calcite, and dolomite, which is consistent with the chemical compositions (Table 2). MgO (0.93–6.2% and 1.13–5.62%, respectively), K_2_O (0.54–4.27% and 1.00–5.36%, respectively), Fe_2_O_3_ (0.06–4.54% and 0.05–8.05%, respectively), and FeO (0.47–2.34% and 0.57–1.90%, respectively) are the second most abundant oxides in the Wufeng and Longmaxi formations. While other oxides including MnO, Na_2_O, P_2_O_5_, and TiO_2_ contents are both lower than 1.0%.

The SiO_2_ content in the carbonaceous marl samples from the Guanyinqiao Formation is relatively high (36.66–58.11%, average of 48.22%). CaO (2.28–18.64%) and Al_2_O_3_ (6.15–14.39%) are the second-most abundant oxides, with averages of 13.09% and 8.51%, respectively. The contents of K_2_O and MgO are 1.81–4.12% and 0.91–3.7%, respectively. The Fe_2_O_3_ content of carbonaceous marls (0.85–12.96%) is relatively higher than the FeO content (0.83–1.26%). The contents of other oxides, such as MnO, Na_2_O, P_2_O_5_, and TiO_2_, are less than 1.0%.

**Figure 5:**
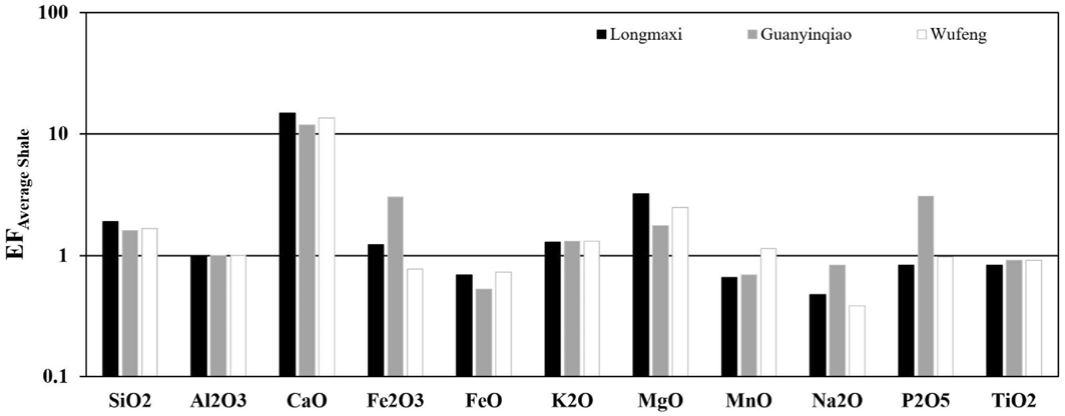
Enrichment factors of the major elements from the Wufeng-Longmaxi formations in the Xindi No. 2 well. The horizontal line is the EF of average shale, which is equal to 1 to highlight the enrichment or depletion of elements.

The enrichment factor(EF) was applied by using aluminium as the detritus index to estimate the enrichment degree of elements in each sample, and the EF value was determined by using the formula: EF_X_ = molar[(X/Al)_sample_/(X/Al)_average shale_], where X represents the element in Table 5. EF_X_ > 1 reflects the relative enrichment of element X, while EF_X_ < 1 indicates that element X is depleted compared to average shale^55,56^. The major element EFs of black shales and carbonaceous marls from the Wufeng-Longmaxi formations are shown in Table 5 and Fig. 5. The enrichment of CaO in the above three formations can be attributed to the existence of calcite (4.0–28%, average of 17.7%) and dolomite (2.3–27.2%,average of 14.3%) according to XRD analysis. The relatively high concentration of magnesium is also related to the dolomite content^57^. As shown in Fig. 5, the K contents of the sediments from the Wufeng, Guanyinqiao, and Longmaxi formations have relatively high K/Al ratios owing to higher illite contents^58^. Titanium, a diagenetically stable constituent of marine sediments, has an EF less than 1.0. The strong correlation between Ti and Al may also be because these two elements are dominated by detrital sources, but not autogenous enrichment^55^.

The chemical index of alteration (CIA) was applied to quantify the degree of chemical weathering^59,60^. The CIA can be calculated using molecular proportions, from the equation CIA = [Al_2_O_3_/(Al_2_O_3_ + CaO^*^ + Na_2_O + K_2_O)] × 100, where CaO^*^ represents the concentration of CaO in silicate minerals only^61^. In this case, the CaO content must be corrected for the presence of carbonates (calcite, dolomite) and apatite. In this study, the available P_2_O_5_ data was initially used for phosphate correction of CaO by the following formula: CaO^*^ = mole CaO − mole P_2_O_5_ × 10/3. If the remaining number of moles was less than that of Na_2_O, the CaO value was adopted as CaO^*^. Otherwise, the CaO value was assumed to be equal to Na_2_O^62^.

#### Rare earth elements

**Figure 6:**
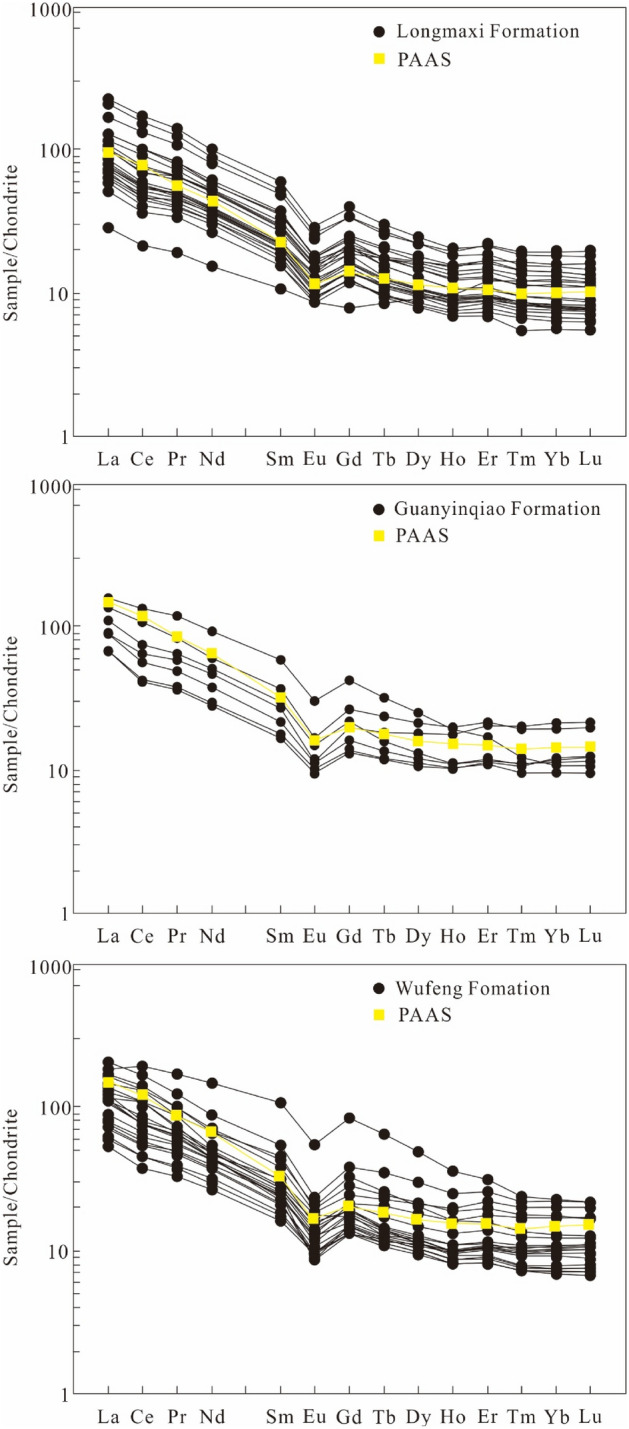
Chondrite-normalized REE distribution patterns of samples from the Wufeng-Longmaxi formations in the Xindi No. 2 well (normalization values are after Taylor and McLennan^64^).

REE analysis and related parameters are listed in Table 3 and shown as chondrite-normalized models in Fig. 6. The samples from all three formations were normalized to chondrite values and the Eu anomaly was calculated according to McLennan^63^, i.e., Eu/Eu^*^ = (Eu_cn_)/[(Sm_sn_ × Gd_cn_)^1/2^], in which the subscript cn denotes normalization of the REE to chondrite values^63^.

**Table 3:** Analytical data of rare earth elements (REEs) of the shales and carbonaceous marls from the Wufeng-Longmaxi formations in the Xindi No. 2 well.

Sample	Height	La	Ce	Pr	Nd	Sm	Eu	Gd	Tb	Dy	Ho	Er	Tm	Yb	Lu	ΣREE	LREE	HREE	LREE/HREE	La_N_/Yb_N_	δEu	δCe
XD2P-B60	2052.48	53.8	105	13.3	47.3	9.2	1.68	8.2	1.13	6.24	1.16	3.54	0.47	3.11	0.46	254.59	230.28	24.31	9.47	12.41	0.58	0.93
XD2P-B59	2053.22	40.3	81.4	10.4	37.7	7.58	1.4	7.07	1.01	5.65	1.04	3.1	0.4	2.6	0.37	200.02	178.78	21.24	8.42	11.12	0.58	0.95
XD2P-B58	2053.92	49.8	94.9	11.9	41.7	8.04	1.5	6.98	0.97	5.58	1.12	3.61	0.5	3.34	0.5	230.44	207.84	22.60	9.20	10.70	0.60	0.92
XD2P-B57	2054.42	30.5	62.4	7.51	26.8	5.25	1.06	4.81	0.66	3.76	0.72	2.11	0.29	1.96	0.28	148.11	133.52	14.59	9.15	11.16	0.63	0.98
XD2P-B56	2055.32	30.9	62.4	7.84	28.4	5.71	1.06	5.25	0.78	4.59	0.89	2.74	0.37	2.4	0.34	153.67	136.31	17.36	7.85	9.24	0.58	0.96
XD2P-B55	2055.92	30.5	62.6	7.83	27.3	5.38	1.05	4.98	0.76	4.13	0.81	2.46	0.34	2.24	0.32	150.70	134.66	16.04	8.40	9.77	0.61	0.97
XD2P-B54	2056.32	27.7	56.2	6.87	24.8	4.89	0.97	4.33	0.67	3.72	0.7	2.16	0.29	1.9	0.28	135.48	121.43	14.05	8.64	10.46	0.63	0.97
XD2P-B53	2056.92	24.2	45.4	6.26	24	4.56	0.92	4.2	0.58	3.25	0.61	1.81	0.24	1.56	0.22	117.81	105.34	12.47	8.45	11.13	0.63	0.88
XD2P-B52	2057.32	23.7	43	5.89	22.2	4.12	0.87	3.87	0.49	2.72	0.5	1.5	0.2	1.33	0.18	110.57	99.78	10.79	9.25	12.78	0.66	0.87
XD2P-B51	2058.22	26	46.9	6.26	23.6	4.41	0.82	4.34	0.59	3.29	0.6	1.76	0.24	1.59	0.23	120.63	107.99	12.64	8.54	11.73	0.57	0.87
XD2P-B50	2058.62	6.86	13.2	1.84	7.21	1.66	0.5	1.63	0.32	2.35	0.55	2.02	0.31	2.14	0.3	40.89	31.27	9.62	3.25	2.30	0.92	0.89
XD2P-B49	2059.58	18.6	34.4	4.71	18.5	3.53	0.65	3.28	0.47	2.7	0.52	1.54	0.21	1.33	0.19	90.63	80.39	10.24	7.85	10.03	0.57	0.88
XD2P-B48	2060.32	20.4	36.3	4.89	18.7	3.46	0.72	3.24	0.46	2.77	0.54	1.59	0.22	1.42	0.21	94.92	84.47	10.45	8.08	10.30	0.65	0.86
XD2P-B47	2061.12	18.2	33.8	4.68	18.5	3.46	0.66	3.34	0.47	2.65	0.51	1.53	0.21	1.38	0.19	89.58	79.30	10.28	7.71	9.46	0.59	0.88
XD2P-B46	2061.92	18.1	33.7	4.67	18.1	3.44	0.66	3.32	0.49	2.72	0.53	1.65	0.22	1.42	0.21	89.23	78.67	10.56	7.45	9.14	0.59	0.88
XD2P-B45	2062.02	17.9	33.5	4.67	18	3.33	0.65	3.42	0.47	2.68	0.52	1.56	0.22	1.39	0.2	88.51	78.05	10.46	7.46	9.24	0.58	0.88
XD2P-B44	2062.60	18.1	33.4	4.52	17.3	3.17	0.6	2.99	0.43	2.51	0.49	1.48	0.21	1.3	0.2	86.70	77.09	9.61	8.02	9.99	0.59	0.88
XD2P-B43	2063.42	16.2	29.9	4.12	15.8	3	0.59	2.89	0.42	2.47	0.48	1.47	0.2	1.29	0.19	79.02	69.61	9.41	7.40	9.01	0.60	0.87
XD2P-B42	2064.07	15.4	28.4	3.89	15.1	2.94	0.62	2.95	0.41	2.41	0.45	1.41	0.19	1.24	0.18	75.59	66.35	9.24	7.18	8.91	0.64	0.88
XD2P-B40	2064.92	17.4	31.7	4.37	17	3.19	0.65	3.16	0.43	2.5	0.48	1.47	0.2	1.29	0.19	84.03	74.31	9.72	7.65	9.68	0.62	0.87
XD2P-B39	2065.42	19.4	34.9	4.69	17.7	3.33	0.69	3.22	0.45	2.61	0.5	1.57	0.21	1.36	0.2	90.83	80.71	10.12	7.98	10.23	0.64	0.87
XD2P-B38	2066.42	14.9	27.3	3.81	15.1	2.75	0.53	2.61	0.35	2.03	0.39	1.15	0.14	0.96	0.14	72.16	64.39	7.77	8.29	11.13	0.60	0.87
XD2P-B37	2067.82	12.2	22.7	3.25	12.4	2.4	0.5	2.42	0.36	2.18	0.43	1.31	0.18	1.15	0.17	61.65	53.45	8.20	6.52	7.61	0.63	0.87
XD2P-B36	2068.52	14.1	24.9	3.57	14.2	2.62	0.53	2.56	0.36	2.1	0.41	1.22	0.17	1.08	0.16	67.98	59.92	8.06	7.43	9.36	0.62	0.84
XD2P-B35	2069.02	23.8	42.2	6.21	24.5	4.57	0.88	4.59	0.65	3.95	0.76	2.34	0.31	2.09	0.3	117.15	102.16	14.99	6.82	8.17	0.58	0.83
XD2P-B34	2069.37	17.1	28.6	4.28	16.8	3.47	0.79	3.88	0.65	4.24	0.87	2.83	0.42	2.71	0.41	87.05	71.04	16.01	4.44	4.53	0.66	0.80
XD2P-B33	2069.52	16	25.2	3.59	13.7	2.69	0.59	2.88	0.44	2.73	0.57	1.87	0.27	1.89	0.29	72.71	61.77	10.94	5.65	6.07	0.64	0.78
XD2P-B32	2069.67	21.6	34.4	4.69	17.6	3.29	0.65	3.34	0.5	3	0.62	1.96	0.28	1.92	0.29	94.14	82.23	11.91	6.90	8.07	0.59	0.80
XD2P-B31	2069.77	37.9	82.5	11.3	43.3	9.06	1.76	8.64	1.2	6.35	1.07	2.81	0.31	1.82	0.27	208.29	185.82	22.47	8.27	14.94	0.60	0.97
XD2P-B30	2069.87	16.2	25.7	3.46	13.2	2.55	0.55	2.72	0.44	2.8	0.58	1.93	0.28	1.99	0.31	72.71	61.66	11.05	5.58	5.84	0.63	0.80
XD2P-B29	2069.94	26.4	45.5	6.16	23.9	4.56	0.86	4.51	0.59	3.27	0.62	1.82	0.24	1.61	0.24	120.28	107.38	12.90	8.32	11.76	0.57	0.84
XD2P-B28	2069.98	32.2	65	7.93	28.2	5.63	0.95	5.47	0.88	5.35	1.09	3.5	0.49	3.27	0.5	160.46	139.91	20.55	6.81	7.06	0.52	0.97
XD2P-B27	2070.32	21.1	39.4	5.63	22	4.16	0.67	4.17	0.68	4.6	1	3.41	0.51	3.65	0.54	111.52	92.96	18.56	5.01	4.15	0.49	0.87
XD2P-B26	2071.22	12.5	22.9	3.12	12.4	2.46	0.53	2.71	0.43	2.79	0.56	1.78	0.24	1.59	0.24	64.25	53.91	10.34	5.21	5.64	0.62	0.87
XD2P-B25	2072.17	14.5	27.6	3.49	13.6	2.66	0.55	2.7	0.4	2.32	0.46	1.34	0.18	1.15	0.17	71.12	62.40	8.72	7.16	9.04	0.62	0.92
XD2P-B24	2072.92	16.7	33.8	4.43	17.7	3.23	0.52	3.14	0.45	2.63	0.48	1.45	0.19	1.25	0.18	86.15	76.38	9.77	7.82	9.58	0.49	0.94
XD2P-B23	2073.55	21	41.1	5.3	20.3	3.58	0.52	3.2	0.43	2.42	0.45	1.35	0.19	1.24	0.18	101.26	91.80	9.46	9.70	12.15	0.46	0.93
XD2P-B22	2074.20	16.8	33.1	4.41	17.2	3.17	0.51	3.11	0.43	2.37	0.46	1.37	0.18	1.2	0.18	84.49	75.19	9.30	8.08	10.04	0.49	0.92
XD2P-B21	2074.77	18.2	35.4	4.56	17.6	3.11	0.62	3.05	0.45	2.57	0.49	1.52	0.2	1.27	0.19	89.23	79.49	9.74	8.16	10.28	0.61	0.93
XD2P-B20	2075.62	27.6	65.7	8.4	31.3	6.97	1.2	6.61	0.95	5.08	0.91	2.6	0.34	2.18	0.32	160.16	141.17	18.99	7.43	9.08	0.53	1.05
XD2P-B19	2076.42	18.8	37.4	5.19	20.5	3.84	0.64	3.73	0.52	2.97	0.56	1.66	0.23	1.53	0.22	97.79	86.37	11.42	7.56	8.81	0.51	0.91
XD2P-B18	2077.1	17.4	33.9	4.74	18.7	3.71	0.68	3.72	0.51	2.85	0.52	1.49	0.2	1.32	0.2	89.94	79.13	10.81	7.32	9.46	0.55	0.90
XD2P-B17	2077.7	25.9	46.3	6.04	22.9	4.17	0.77	4.3	0.64	3.73	0.74	2.3	0.32	2.08	0.31	120.50	106.08	14.42	7.36	8.93	0.55	0.88
XD2P-B16	2078.4	26.3	45.8	5.8	20.5	3.43	0.73	3.24	0.48	2.81	0.56	1.74	0.24	1.6	0.24	113.47	102.56	10.91	9.40	11.79	0.66	0.87
XD2P-B15	2078.79	28.4	49.1	6.51	23.3	4.28	0.81	4	0.53	3.12	0.61	1.85	0.26	1.73	0.25	124.75	112.40	12.35	9.10	11.78	0.59	0.85
XD2P-B14	2079.38	26.6	52.2	6.33	20.4	3.99	0.73	3.67	0.54	3.18	0.61	1.89	0.27	1.84	0.28	122.53	110.25	12.28	8.98	10.37	0.57	0.95
XD2P-B13	2079.67	13.9	27.5	3.7	14.7	2.84	0.53	2.89	0.45	2.74	0.54	1.78	0.26	1.81	0.27	73.91	63.17	10.74	5.88	5.51	0.56	0.92
XD2P-B12	2080.3	40.6	85.8	9.59	31.4	5.82	1.07	5.01	0.85	5.3	1.1	3.63	0.51	3.38	0.5	194.56	174.28	20.28	8.59	8.62	0.59	1.03
XD2P-B10	2080.57	43.8	118	16.2	68.5	16.4	3.17	17.1	2.45	12.4	2.02	5.18	0.6	3.86	0.55	310.23	266.07	44.16	6.03	8.14	0.57	1.09
XD2P-B8	2080.7	49	103	11.7	41	8.33	1.36	7.87	1.31	7.6	1.41	4.21	0.56	3.74	0.55	241.64	214.39	27.25	7.87	9.40	0.51	1.02
XD2P-B6	2080.99	34.3	81.5	9.26	32.8	6.54	1.14	5.8	0.91	5.43	1.06	3.25	0.45	2.97	0.42	185.83	165.54	20.29	8.16	8.28	0.55	1.10
XD2P-B5	2081.32	30.3	66.6	6.74	22	4.78	0.87	4.32	0.76	4.57	0.9	2.92	0.42	2.86	0.43	148.47	131.29	17.18	7.64	7.60	0.57	1.09
XD2P-B4	2081.77	32.6	67.3	6.96	22.1	4.38	0.91	3.86	0.53	3	0.56	1.81	0.25	1.75	0.27	146.28	134.25	12.03	11.16	13.36	0.66	1.04
XD2P-B3	2081.82	38.9	80.5	8.14	25.3	4.68	1	3.59	0.5	2.81	0.53	1.73	0.24	1.71	0.26	169.89	158.52	11.37	13.94	16.32	0.72	1.05

The total rare earth element contents (∑REEs) from the Wufeng, Guanyinqiao and Longmaxi formations vary significantly from 40.89 to 310.23 ppm, with average values of 133.16 ppm, 111.52 ppm and 113 ppm (Table 3), which are all lower than the average value of Post-Archean Australian Shale (PAAS) (184.77 ppm)^63^. Similar REE distribution curves are shown for all three formations, including relatively light rare earth element (LREE) enrichment, flat heavy rare earth element (HREE) patterns, and negative Eu anomalies (Fig. 6). The contents of LREEs (53.91–266.07 ppm) from the above three formations are significantly higher than those of HREEs (7.77–44.16 ppm). Similar conclusions can be drawn based on the ratios of La_cn_/Yb_cn_, where La_cn_/Yb_cn_ < 1(> 1) shows HREE enrichment (depletion) compared to LREEs. The ratios La_cn_/Yb_cn_ from the three formations change from 3.25 to 16.32, with an average of 8.17 (Table 3). All samples from the Wufeng-Longmaxi formations exhibit negative Eu anomalies, varying from 0.46 to 0.97, with a median of 0.68, which is similar to the Eu anomalies of the PAAS (δEu = 0.66).

#### Trace elements

Analytical results of trace elements are given in Table 4. The elements Ba (with an average of 1444.48 μg/g, 1933.71 μg/g, and 2175.54 μg/g, respectively), Sr (with an average of 323.51 μg/g, 263.43 μg/g, and 228.98 μg/g, respectively), V (with an average of 138.60 μg/g, 135.63 μg/g, and 183.81 μg/g, respectively), Zn (with an average of 348.87 μg/g, 47.37 μg/g, and 127.71 μg/g, respectively) in Wufeng, Guanyinqiao and Longmaxi formations are the most abundant. Ni (with averages of 52.81 μg/g, 104.26 μg/g, 79.63 μg/g, respectively) and Zr (with averages of 85.83 μg/g, 139.23 μg/g, and 68.27 μg/g, respectively) are the second most abundant elements.

**Table 4:** Analytical data of some trace elements from the Wufeng-Longmaxi formations in the Xindi No. 2 well.

Sample	XD2P-B60	XD2P-B59	XD2P-B58	XD2P-B57	XD2P-B56	XD2P-B55	XD2P-B54	XD2P-B53	XD2P-B52	XD2P-B51	XD2P-B50	XD2P-B49	XD2P-B48	XD2P-B47
Height (m)	2052.48	2053.22	2053.92	2054.42	2055.32	2055.92	2056.32	2056.92	2057.32	2058.22	2058.62	2059.58	2060.32	2061.12
Co	14.2	13.5	9.35	11.7	9.55	8.99	9.51	8.65	9.76	5.98	9.02	6.49	6.8	5.95
Ni	97.3	110	99.8	80	77.8	75	68.6	68.5	67.6	56	70.4	59.7	66.6	63.2
Cu	70.6	69.4	59.7	52.3	48.1	44.2	42.9	45.2	44.7	26.5	31.9	32.4	28.9	27.8
Zn	33.9	188	82.3	17	23.3	141	40.3	43	26.4	41.2	176	362	43.1	104
Ga	18	15.8	15.5	13	12	11.6	10.7	9.41	10.2	8.02	17.5	7.67	8.8	7.57
Rb	166	152	143	126	115	110	103	90.6	97.4	74.1	173	74.3	83.7	73.6
Sr	116	134	160	220	238	236	286	270	215	331	106	323	316	280
Mo	47.1	62.1	48.8	40.9	48.1	43.5	44.8	51.3	47.8	37.1	37	39.9	43.8	44.8
Cd	0.41	2.32	1.24	0.41	0.44	2.45	0.65	0.71	0.46	0.52	1.49	2.77	0.46	0.87
Cs	11.3	9.84	9.19	7.72	6.8	6.84	6.35	5.54	5.79	4.39	12.7	4.25	4.87	4.27
Ba	3898	3476	4068	3339	3238	2607	2295	2210	3545	1867	3470	1760	1902	1533
Pb	24.3	21.7	18.5	20.2	14.5	13	12.5	11.8	14.5	8.7	47.3	9.09	10	9.27
Th	14.5	14.2	13.4	11.7	12.1	11.2	11	9.4	10.2	7.37	34.3	7.44	8.39	7.35
U	19.8	21.1	18.3	13.5	14.9	14.1	12	12.3	11	9.99	13.1	11.8	10.2	11
Nb	17.7	11.5	12.7	9.43	9.38	8.78	7.89	6.99	7.95	7.32	6.51	5.47	6.37	5.63
Ta	0.93	0.85	0.87	0.76	0.72	0.67	0.63	0.56	0.62	0.46	1.79	0.44	0.51	0.46
Zr	116	92.8	139	67.9	74.7	103	69.2	55.8	56.8	49.6	172	49.6	50.1	56.1
Hf	2.83	2.72	3.35	2.38	2.65	2.64	2.44	1.98	2.05	1.73	10.1	1.7	1.74	1.89
Ti	3004	2802	2493	2370	2323	2159	2044	1825	2070	1394	1897	1462	1691	1468
V	188	211	158	136	160	143	145	136	118	106	136	117	128	150
Cr	57.9	53.7	46.4	42.5	41.9	38.8	36.7	32.7	35.6	24.7	18.6	25.8	29.4	26.7
Sc	11.1	10.8	10.4	8.13	8.8	8.19	7.78	6.21	6.15	5.19	7.8	5.43	6	5.33
La/Th	3.71	2.84	3.72	2.61	2.55	2.72	2.52	2.57	2.32	3.53	0.20	2.50	2.43	2.48
Th/Sc	1.31	1.31	1.29	1.44	1.38	1.37	1.41	1.51	1.66	1.42	4.40	1.37	1.40	1.38
Zr/Sc	10.45	8.59	13.37	8.35	8.49	12.58	8.89	8.99	9.24	9.56	22.05	9.13	8.35	10.53
U-EF	31.84	38.18	33.37	29.00	35.94	34.53	32.88	37.09	29.08	35.12	13.38	45.11	33.09	42.44
Mo-EF	68.17	101.12	80.08	79.07	104.43	95.88	110.47	139.22	113.71	117.38	34.01	137.28	127.87	155.56
U/Th	1.37	1.49	1.37	1.15	1.23	1.26	1.09	1.31	1.08	1.36	0.38	1.59	1.22	1.50
V/V + Ni	0.66	0.66	0.61	0.63	0.67	0.66	0.68	0.67	0.64	0.65	0.66	0.66	0.66	0.70
Ni/Co	6.85	8.15	10.67	6.84	8.15	8.34	7.21	7.92	6.93	9.36	7.80	9.20	9.79	10.62
V/Cr	3.25	3.93	3.41	3.20	3.82	3.69	3.95	4.16	3.31	4.29	7.31	4.53	4.35	5.62

Sample	XD2P-B46	XD2P-B45	XD2P-B44	XD2P-B43	XD2P-B42	XD2P-B40	XD2P-B39	XD2P-B38	XD2P-B37	XD2P-B36	XD2P-B35	XD2P-B34	XD2P-B33	XD2P-B32
Height (m)	2061.92	2062.02	2062.60	2063.42	2064.07	2064.92	2065.42	2066.42	2067.82	2068.52	2069.02	2069.37	2069.52	2069.67
Co	6.06	5.92	6.27	5.68	5.73	5.72	6.24	5.03	3.5	4.65	6.16	3.66	4.43	10.9
Ni	59.5	67.5	59.9	58.4	62.4	63.1	77.8	103	74.3	105	139	140	59.9	81.4
Cu	24.3	28	28.6	23.3	25	24	29.6	40.7	45.1	36.5	64.2	117	25.7	25.2
Zn	61.7	64.8	66.4	76.6	69.4	71.2	37.7	88.2	101	815	305	242	15.2	14
Ga	7.12	6.92	8.51	7.36	6.78	7.56	8.81	6.56	4.71	6.34	8.8	5.32	9.56	8.35
Rb	69.7	66.4	83	70.9	62.6	72.9	85.8	60.8	39.3	48.9	75	38.8	88.1	73.4
Sr	314	281	239	268	293	319	242	79.9	129	72.8	93.9	391	306	353
Mo	38.7	45.8	47.6	34.7	34	38.8	49.4	61	45.5	59.1	111	88.9	3.16	3.74
Cd	0.54	0.65	0.64	0.72	0.62	0.73	0.51	0.98	1.02	7.97	4.91	4.81	0.13	0.19
Cs	4.09	3.72	4.78	3.89	3.69	3.97	4.79	3.36	2.33	2.96	4.09	2.01	4.82	3.64
Ba	1516	1553	1640	1427	1374	1381	1499	1583	1283	1441	1623	1036	1668	1488
Pb	8.31	8.69	10.5	9.8	9.1	8.76	9.63	7.87	5.8	8.01	13.8	8.67	9.29	21
Th	7.05	6.55	8.4	7.36	6.43	7.17	8.29	6.13	4.34	5.29	7.93	4.64	8.5	7.49
U	10.7	13.7	11.7	10.7	8.87	10.3	13.4	18	12.5	16.2	35.3	64.5	5.91	2.27
Nb	5.46	5.2	6.94	5.97	5.29	5.46	6.63	4.69	3.6	4.17	5.91	3.97	8.2	6.61
Ta	0.45	0.41	0.54	0.48	0.4	0.44	0.54	0.39	0.29	0.35	0.52	0.28	0.66	0.53
Zr	54.4	48.5	67.4	56	45.2	53.8	63.9	45	35.1	34.9	56.3	61.9	66.7	80.9
Hf	1.85	1.64	2.37	1.91	1.52	1.87	2.14	1.55	1.21	1.23	1.96	1.92	2.46	2.85
Ti	1402	1363	1670	1427	1344	1436	1722	1213	789	1095	1465	810	2024	1762
V	136	131	166	159	130	167	213	248	190	320	484	403	122	62.4
Cr	24.6	24	29.7	25.9	24	26.8	32.3	26.4	19.2	26.6	40.4	47.4	40.8	27.5
Sc	5.22	4.77	5.53	5	4.87	5.22	5.7	4.22	3.59	3.99	5.77	4.7	9.09	7.59
La/Th	2.57	2.73	2.15	2.20	2.40	2.43	2.34	2.43	2.81	2.67	3.00	3.69	1.88	2.88
Th/Sc	1.35	1.37	1.52	1.47	1.32	1.37	1.45	1.45	1.21	1.33	1.37	0.99	0.94	0.99
Zr/Sc	10.42	10.17	12.19	11.20	9.28	10.31	11.21	10.66	9.78	8.75	9.76	13.17	7.34	10.66
U-EF	43.86	58.44	39.35	43.10	38.07	39.81	45.51	76.32	75.60	74.40	123.89	399.32	18.03	7.75
Mo-EF	142.77	175.84	144.09	125.81	131.33	134.97	150.99	232.77	247.68	244.27	350.61	495.35	8.68	11.49
U/Th	1.52	2.09	1.39	1.45	1.38	1.44	1.62	2.94	2.88	3.06	4.45	13.90	0.70	0.30
V/V + Ni	0.70	0.66	0.73	0.73	0.68	0.73	0.73	0.71	0.72	0.75	0.78	0.74	0.67	0.43
Ni/Co	9.82	11.40	9.55	10.28	10.89	11.03	12.47	20.48	21.23	22.58	22.56	38.25	13.52	7.47
V/Cr	5.53	5.46	5.59	6.14	5.42	6.23	6.59	9.39	9.90	12.03	11.98	8.50	2.99	2.27

Sample	XD2P-B31	XD2P-B30	XD2P-B29	XD2P-B28	XD2P-B27	XD2P-B26	XD2P-B25	XD2P-B24	XD2P-B23	XD2P-B22	XD2P-B21	XD2P-B20	XD2P-B19	XD2P-B18
Height (m)	2069.77	2069.87	2069.94	2069.98	2070.32	2071.22	2072.17	2072.92	2073.55	2074.20	2074.77	2075.62	2076.42	2077.1
Co	7.96	8.22	11.2	26.9	4.8	6.99	6.19	6.14	10.8	7.02	5.77	11.7	6.29	7.37
Ni	84.9	60.6	108	208	127	48.9	42.6	44.9	76.2	53.2	34.5	56.8	55.4	71.8
Cu	58.6	20.4	51.3	91	41.1	23.8	34.4	33.9	65.8	42.7	33.7	56.9	61.3	114
Zn	77.8	19.2	55.8	69.2	80.4	6.48	9.75	9.82	72.9	87.5	33	17.7	120	210
Ga	11.2	8.56	12.2	11.6	22.5	2.58	5.11	6.71	9.68	6.96	6.97	10	7.25	7.7
Rb	87.5	77.1	98.3	95.2	162	20.2	44.7	66.7	91.5	68.4	70.9	96.4	73.4	75.4
Sr	103	402	157	339	184	521	900	358	156	294	365	355	119	121
Mo	3.33	1.75	9.43	71.8	32.6	18.1	8.55	7.95	14.2	9.09	5.36	15	6.98	15.5
Cd	1.29	0.27	0.89	1.38	1.31	0.13	0.08	0.05	0.53	0.55	0.15	0.11	0.54	0.98
Cs	3.41	3.85	4.08	5.19	8.23	1.09	2.38	3.39	4.97	3.46	3.42	5.01	3.59	3.71
Ba	1767	1460	1822	1569	3762	472	878	1046	1325	993	1050	1389	1143	1208
Pb	30.6	17.5	29.6	27.1	17.9	12.9	21.5	20.9	38.8	21.9	18.6	70.7	28.2	33
Th	8.89	6.98	9.78	10.6	24	2.77	4.67	6.29	9.44	6.61	6.56	10.2	7.24	7.51
U	2.62	2.34	4.38	26.3	12.6	6.36	5.33	5.14	7.28	5.83	4.96	5.68	5.57	7.89
Nb	8.61	6.13	10.3	11.1	15	2.71	3.9	5.49	7.75	5.43	10	17	5.33	5.62
Ta	0.64	0.52	0.73	0.8	1.46	0.23	0.33	0.39	0.58	0.44	0.61	1.28	0.46	0.49
Zr	238	71	208	130	180	36.8	34.4	45.1	68.7	45.8	49.5	101	45.9	47.6
Hf	6.63	2.44	5.75	3.32	5.62	1.19	1.18	1.5	2.34	1.52	1.58	3.13	1.63	1.69
Ti	2669	1690	2910	2362	2075	535	890	1318	1967	1439	1597	3139	1483	1549
V	59.3	59.7	104	234	308	50.9	112	150	200	145	118	82	174	329
Cr	36.5	27.3	39.2	46.8	39.5	10.2	31.3	39.9	66.4	44.2	35.9	31.1	44.5	53.5
Sc	5.47	10.1	7.61	9.89	11.7	2.68	4.33	5.59	6.94	5.38	5.07	6.27	5.95	6.84
La/Th	4.26	2.32	2.70	3.04	0.88	4.51	3.10	2.66	2.22	2.54	2.77	2.71	2.60	2.32
Th/Sc	1.63	0.69	1.29	1.07	2.05	1.03	1.08	1.13	1.36	1.23	1.29	1.63	1.22	1.10
Zr/Sc	43.51	7.03	27.33	13.14	15.38	13.73	7.94	8.07	9.90	8.51	9.76	16.11	7.71	6.96
U-EF	6.19	7.85	9.82	71.87	18.38	72.94	29.67	17.83	21.43	18.62	19.53	16.79	20.99	27.37
Mo-EF	7.08	5.28	19.03	176.59	42.79	186.82	42.84	24.82	37.62	26.13	19.00	39.91	23.67	48.39
U/Th	0.29	0.34	0.45	2.48	0.53	2.30	1.14	0.82	0.77	0.88	0.76	0.56	0.77	1.05
V/V + Ni	0.41	0.50	0.49	0.53	0.71	0.51	0.72	0.77	0.72	0.73	0.77	0.59	0.76	0.82
Ni/Co	10.67	7.37	9.64	7.73	26.46	7.00	6.88	7.31	7.06	7.58	5.98	4.85	8.81	9.74
V/Cr	1.62	2.19	2.65	5.00	7.80	4.99	3.58	3.76	3.01	3.28	3.29	2.64	3.91	6.15

Sample	XD2P-B17	XD2P-B16	XD2P-B15	XD2P-B14	XD2P-B13	XD2P-B12	XD2P-B10	XD2P-B8	XD2P-B6	XD2P-B5	XD2P-B4	XD2P-B3		
Height(m)	2077.7	2078.4	2078.79	2079.38	2079.67	2080.3	2080.57	2080.7	2080.99	2081.32	2081.77	2081.82		
Co	9.21	8.47	8.82	5.48	6.79	8.11	7.16	7.14	13.9	16.8	6.13	7.91		
Ni	53	49.2	67.1	42.7	36.2	54.2	48.3	42.8	69	58.5	44.7	59.1		
Cu	61.1	68.5	100	74.3	57.7	97	55.7	48.4	92.3	56.6	36.5	51.3		
Zn	19.1	24.3	27.7	102	28.4	305	4942	847	371	34.5	27.7	30.4		
Ga	11.7	12.1	11.9	9.46	6.39	19.3	19.5	14.6	15.9	14.3	18.7	20.3		
Rb	116	122	123	97	66	176	159	134	145	127	172	180		
Sr	480	378	158	140	53.8	141	383	444	360	391	420	256		
Mo	3.84	2.39	3.01	3.23	10.3	0.47	0.61	0.33	0.36	0.3	0.16	0.19		
Cd	0.05	0.05	0.05	0.33	0.09	0.55	7.97	1.45	0.7	0.05	0.05	0.05		
Cs	5.96	6.3	6.4	4.79	3.15	9.78	8.54	6.89	7.83	6.64	9.12	9.85		
Ba	1521	1429	1528	1501	1492	2124	2157	1663	1564	1398	2125	2328		
Pb	39.3	25	28.1	22	21.7	39.9	24.8	75.7	109	25.1	13.2	29.9		
Th	9.6	11.3	12.4	9.65	8.44	16.4	12.7	11.8	14.6	14.9	18.9	21.3		
U	6.53	4.48	4.73	4.34	6.97	3.47	3.84	3.09	2.97	2.73	2.95	3.46		
Nb	8.76	9.52	9.8	9.53	5.56	18.9	12.8	12.3	12.9	16.7	15	17.8		
Ta	0.65	0.77	0.79	0.64	0.56	1.11	0.78	0.78	0.95	1.31	1.16	1.33		
Zr	66.8	67.2	92.5	62.3	58.8	172	143	126	133	123	136	147		
Hf	2.16	2.42	2.76	2.03	2.53	4.2	3.19	2.71	3.27	3.79	4.02	4.48		
Ti	2128	2484	2576	1909	1809	3119	2680	2562	3149	3131	3890	4499		
V	99.7	150	112	130	176	147	144	124	160	102	94.1	111		
Cr	57.5	63.8	67.1	48.9	29.1	63.9	50.7	48.2	62.9	45.9	65.6	81.5		
Sc	8.95	9.58	9.1	7.93	6.13	14.1	16.6	13.6	14.3	11.7	15.4	14.4		
La/Th	2.70	2.33	2.29	2.76	1.65	2.48	3.45	4.15	2.35	2.03	1.72	1.83		
Th/Sc	1.07	1.18	1.36	1.22	1.38	1.16	0.77	0.87	1.02	1.27	1.23	1.48		
Zr/Sc	7.46	7.01	10.16	7.86	9.59	12.20	8.61	9.26	9.30	10.51	8.83	10.21		
U-EF	15.77	10.75	11.07	11.88	25.31	5.19	6.27	6.28	5.41	5.45	4.57	4.97		
Mo-EF	8.35	5.16	6.34	7.95	33.66	0.63	0.90	0.60	0.59	0.54	0.22	0.25		
U/Th	0.68	0.40	0.38	0.45	0.83	0.21	0.30	0.26	0.20	0.18	0.16	0.16		
V/V + Ni	0.65	0.75	0.63	0.75	0.83	0.73	0.75	0.74	0.70	0.64	0.68	0.65		
Ni/Co	5.75	5.81	7.61	7.79	5.33	6.68	6.75	5.99	4.96	3.48	7.29	7.47		
V/Cr	1.73	2.35	1.67	2.66	6.05	2.30	2.84	2.57	2.54	2.22	1.43	1.36		

**Figure 7:**
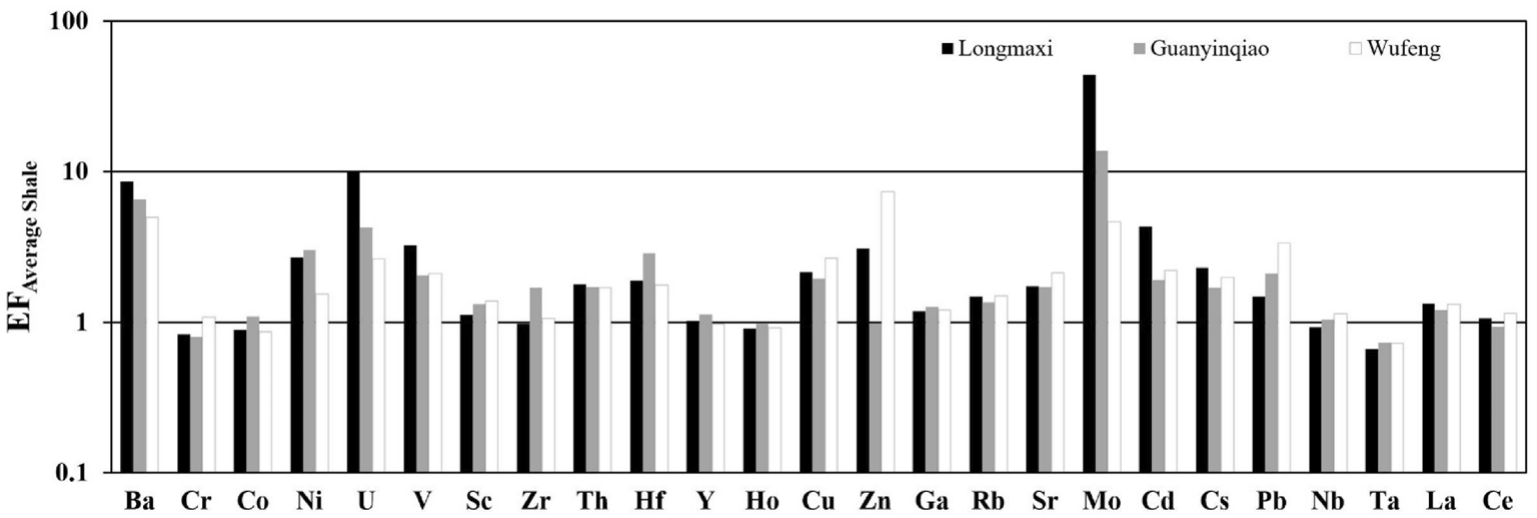
The EFs of trace elements relative to average shale in the Xindi No. 2 well. The horizontal line is the EF of average shale, which is equal to 1 to highlight the enrichment or depletion of elements.

The average contents of all other elements are generally below 50 μg/g. The EFs for the selected trace elements of the three formations are shown in Fig. 5B and Table 5. Some trace elements in the Wufeng, Guanyinqiao, and Longmaxi formations show significant enrichment, including Ba (EF = 4.95, 6.45 and 8.57, respectively), U (EF = 2.64, 4.26 and 9.92, respectively), V (EF = 2.12, 2.05 and 3.23, respectively), Mo (EF = 4.64, 13.76 and 12.84, respectively), Hf (EF = 1.78, 2.88 and 1.91, respectively), Ni (EF = 1.54, 3.01 and 2.67, respectively), and Th (EF = 1.71, 1.73 and 1.79, respectively). Other elements Ho (EF = 0.92, 0.98 and 0.92 respectively), Ta (EF = 0.73, 0.74 and 0.66, respectively), Cr (EF = 1.09, 0.80 and 0.84, respectively) and Co (EF = 0.87, 1.10 and 0.90, respectively) have average EFs less than or approximately equal to 1.0 (Fig. 7). Most of the remaining trace elements in these three formations show slight enrichment. The trace element variation patterns in all three formations are similar (Fig. 7).

**Table 5:** Average concentrations of all elements normalized to Al from the Wufeng-Longmaxi formations in the Xindi No. 2 well.

Oxide/element	Average Shale		Longmaxi			Guanyinqiao			Wufeng		
	Abundance	/Al	n = 26			n = 7			n = 21		
			Abundance	/Al	EF	Abundance	/Al	EF	Abundance	/Al	EF
SiO_2_ (%)	58.90	3.53	49.01	6.71	1.90	48.22	5.66	1.60	49.48	5.88	1.67
Al_2_O_3_ (%)	16.70	[1]	7.31	[1]	1.00	8.51	[1]	1.00	8.41	[1]	1.00
CaO (%)	2.20	0.13	14.04	1.92	14.77	13.09	1.54	11.83	14.74	1.75	13.48
Fe_2_O_3_ (%)	2.80	0.17	1.52	0.21	1.23	4.35	0.51	3.00	1.10	0.13	0.77
FeO (%)	3.70	0.22	1.11	0.15	0.69	0.99	0.12	0.53	1.35	0.16	0.73
K_2_O (%)	3.60	0.22	2.07	0.28	1.29	2.46	0.29	1.31	2.43	0.29	1.31
MgO (%)	2.60	0.16	3.72	0.51	3.18	2.40	0.28	1.76	3.33	0.40	2.47
MnO (%)	0.09	0.01	0.05	0.01	0.66	0.06	0.01	0.69	0.10	0.01	1.14
Na_2_O (%)	1.60	0.10	0.35	0.05	0.48	0.71	0.08	0.83	0.32	0.04	0.38
P_2_O_5_ (%)	0.16	0.01	0.06	0.01	0.83	0.26	0.03	3.05	0.08	0.01	0.97
TiO_2_ (%)	0.78	0.05	0.30	0.04	0.83	0.39	0.05	0.91	0.38	0.05	0.91
Ba (μg/g)	580.00	65.60	2175.54	562.24	8.57	1933.71	429.21	6.54	1444.48	324.43	4.95
Cr (μg/g)	90.00	10.20	33.03	8.54	0.84	36.80	8.17	0.80	49.62	11.15	1.09
Co (μg/g)	19.00	2.15	7.46	1.93	0.90	10.63	2.36	1.10	8.29	1.86	0.87
Ni (μg/g)	68.00	7.70	79.63	20.58	2.67	104.26	23.14	3.01	52.81	11.86	1.54
U (μg/g)	3.70	0.42	16.11	4.16	9.92	8.06	1.79	4.26	4.93	1.11	2.64
V (μg/g)	130.00	14.70	183.81	47.50	3.23	135.63	30.10	2.05	138.60	31.13	2.12
Sc (μg/g)	13.00	1.47	6.38	1.65	1.12	8.78	1.95	1.33	9.09	2.04	1.39
Zr (μg/g)	160.00	18.10	68.27	17.64	0.97	139.23	30.90	1.71	85.83	19.28	1.07
Th (μg/g)	12.00	1.40	9.70	2.51	1.79	10.89	2.42	1.73	10.63	2.39	1.71
Hf (μg/g)	2.80	0.32	2.36	0.61	1.91	4.15	0.92	2.88	2.54	0.57	1.78
Y (μg/g)	41.00	4.60	18.23	4.71	1.02	23.53	5.22	1.14	19.97	4.48	0.97
Ho (μg/g)	1.60	0.18	0.64	0.16	0.92	0.79	0.18	0.98	0.74	0.17	0.92
Cu (μg/g)	45.00	5.10	42.73	11.04	2.17	44.76	9.93	1.95	60.28	13.54	2.65
Zn (μg/g)	95.00	10.70	127.71	33.01	3.08	47.37	10.51	0.98	348.87	78.36	7.32
Ga (μg/g)	19.00	2.10	9.64	2.49	1.19	12.00	2.66	1.27	11.29	2.54	1.21
Rb (μg/g)	140.00	15.80	90.61	23.42	1.48	97.37	21.61	1.37	105.93	23.79	1.51
Sr (μg/g)	300.00	33.90	228.98	59.18	1.75	263.43	58.47	1.72	323.51	72.66	2.14
Mo (μg/g)	2.60	0.29	49.67	12.84	44.27	17.97	3.99	13.76	6.00	1.35	4.64
Cd (μg/g)	0.80	0.09	1.51	0.39	4.34	0.78	0.17	1.92	0.89	0.20	2.22
Cs (μg/g)	5.50	0.62	5.52	1.43	2.30	4.75	1.05	1.70	5.54	1.24	2.01
Pb (μg/g)	20.00	2.30	13.24	3.42	1.49	21.86	4.85	2.11	34.30	7.70	3.35
Nb (μg/g)	18.00	2.00	7.19	1.86	0.93	9.42	2.09	1.05	10.13	2.28	1.14
Ta (μg/g)	2.00	0.23	0.59	0.15	0.66	0.76	0.17	0.74	0.74	0.17	0.73
La (μg/g)	40.00	4.50	23.31	6.02	1.34	24.49	5.43	1.21	26.39	5.93	1.32
Ce (μg/g)	95.00	10.70	44.20	11.42	1.07	45.39	10.07	0.94	54.98	12.35	1.15

#### Organic carbon isotopes

Fifty-four samples were analysed for δ^13^C_org_ values across the Ordovician–Silurian boundary succession at the Xindi No. 2 well (Table 2). The δ^13^C_org_ values commonly vary between − 27.8 and − 29.7‰ in *Dicellograptus complanatus*–*Paraorthograptus pacificus* biozones, showing a positive excursion starting from the upper *P. pacificus* zone and reaching the maximum (− 25.3‰) in the uppermost part of the *Metabolograptus. persculptus* zone in the uppermost Hirnantian. Going upwards, the δ^13^C_org_ values decrease rapidly from − 26.3 to − 29.3‰ in the lower part of the *Akidograptus. ascensus* zone in the early Rhuddanian stage. Farther up, the δ^13^C_org_ values are relatively consistent between − 28.5 and − 30.1‰ from the *A.ascensus* through the *Cystograptus. veiculosus* biozones.

## Discussion

### Provenance and tectonic setting

In general, some heavy minerals, such as zircon, are enriched gradually during sedimentary recycling^62^. According to Taylor et al.^64^, the Th/Sc ratios reflect bulk source compositions, because Sc and Th are transferred quantitatively from the source to the sediment. McLennan^63^ suggests that Zr/Sc ratios can be used as tracers for zircon or heavy mineral concentrations, as zirconium is mainly concentrated in zircons, in which less resistant minerals are preferentially destroyed.

**Figure 8:**
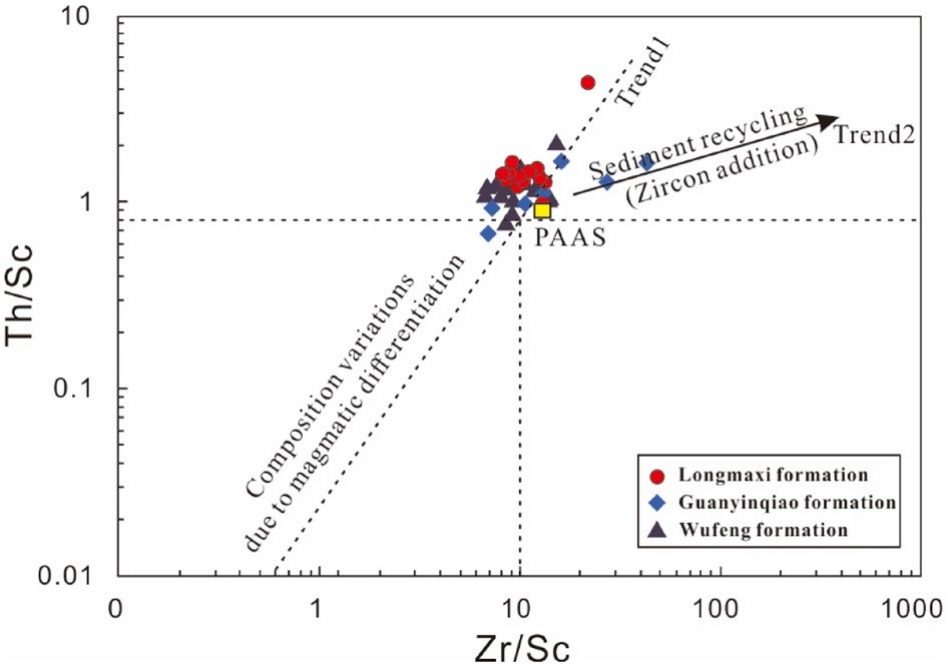
Th/Sc–Zr/Sc bivariate diagram of shales and carbonaceous marls to show their sediment recycling (McLennan et al.^62^).

Therefore, the Th/Sc and Zr/Sc ratios in this study and their bivariate plots were used to derive their sediment recycling degrees and mineral composition varieties^63,64^. The Th/Sc and Zr/Sc ratios of these three formations fluctuated from 0.69 to 4.39 (average of 1.34) and from 5.64 to 43.51 (average of 9.76, Table 4). Both of these ratios indicate a lower fractionation, and the composition is close to PAAS. These data combined with the Th/Sc-Zr/Sc bivariate diagram (Fig. 8) indicate that during the deposition of the Wufeng-Longmaxi formations, sedimentary recycling was minor, and few clastic components came from older sediments. Furthermore, the sedimentary recycling of the studied samples is low, indicating that these geochemical data can be used to identify the provenance.

**Figure 9:**
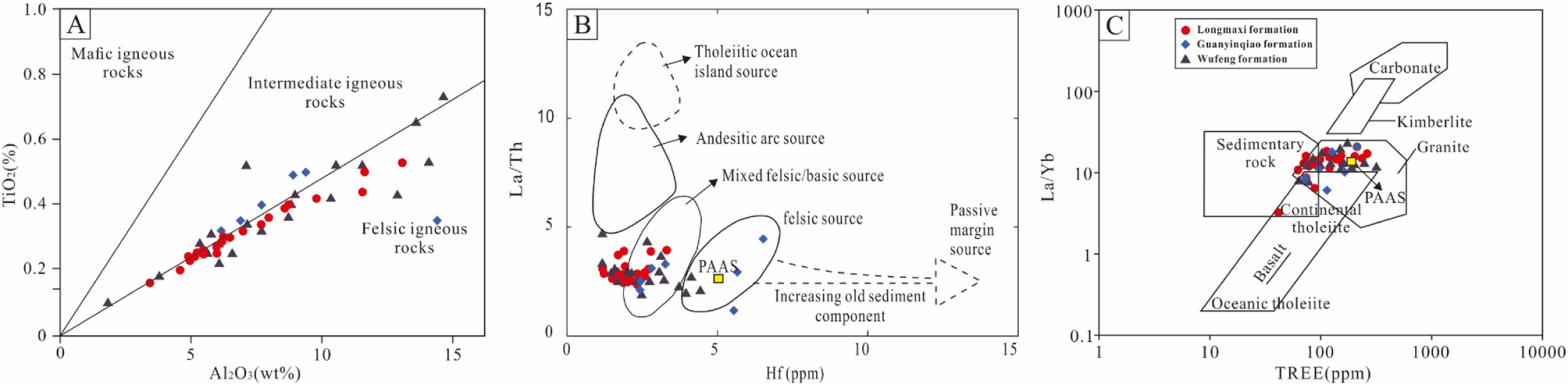
Provenance identification plots for the studied samples. (**A**) Al_2_O_3_ vs. TiO_2_ (after Hayashi et al.^69^); (**B**) La/Th vs. Hf (after Floyd and Leveridge^67^); (**C**) La/Yb vs ∑REE (after Roser and Korsch^68^).

Geochemical data of detrital sediments have stable geochemical properties during weathering, transportation, and diagenesis; therefore, they can provide reliable information on provenance^65,66^. To infer the provenance of sedimentary rocks, several authors have proposed major-based (e.g., Al_2_O_3_, TiO_2_, and K_2_O) discrimination diagrams in various studies of unknown basins^67,68^. According to Hayashi et al.^69^, the Al_2_O_3_/TiO_2_ ratio change in igneous rocks is as follows: (a) the values of Al_2_O_3_/TiO_2_ in mafic igneous rocks vary from 3 to 8, (b) the values of Al_2_O_3_/TiO_2_ in intermediate igneous rocks fluctuate from 8 to 21, and (c) the values of Al_2_O_3_/TiO_2_ in felsic rocks vary from 21 to 70. The Al_2_O_3_/TiO_2_ ratio of the shales in the Wufeng Formation varies from 13.65 to 41.11 with an average of 22.11, the Al_2_O_3_/TiO_2_ ratio of the carbonaceous marls in the Guanyinqiao Formation varies from 18.12 to 41.11 with a median of 19.22, and the Al_2_O_3_/TiO_2_ ratio of the shales in the Longmaxi Formation varies from 20.375 to 60.44 (average of 23.59). These values suggest that the source rocks of the above formation are mostly between intermediate and felsic igneous rocks (Fig. 9A).

REE distributions in sedimentary rocks have played a vital role. The stable and unaffected properties during weathering, erosion, and early diagenesis make them valuable to provenance, leading to their special utility in tracing the source of sedimentary rocks^70,71^. The Eu anomalies in sediments are generally considered to have been inherited from the source rocks^72–74^. Generally, the LREE/HREE ratio of mafic rocks is low, and there is no Eu anomaly, while the LREE/HREE ratio of felsic rocks is usually high, and the Eu anomaly is significant^75,76^. The normalized abundance and pattern of chondrites indicate that all samples are characterized by LREE enrichment, HREE deficits, and distinctly negative Eu anomalies (0.46–0.92; average of 0.6). All of these results suggest that felsic source rocks are the major source rocks for the Wufeng-Longmaxi formations.

In addition, several stable elements (e.g., La, Th, Hf, Yb, Zr, REEs) have been used to deduce the provenance of sedimentary rocks due to their immobility during sedimentation^76–78^. On the bivariate diagram of La/Th-Hf^67^, most samples plot in or near the felsic source field (Fig. 9B). In addition, the bivariate diagram of La/Yb vs. ∑REE reflects that the Wufeng-Longmaxi samples mainly plot in the granite source rocks (Fig. 9C). Overall, the provenance discrimination diagrams reveal that felsic (granitic) source rocks are the major source rocks for the studied Wufeng-Longmaxi formations.

**Figure 10:**
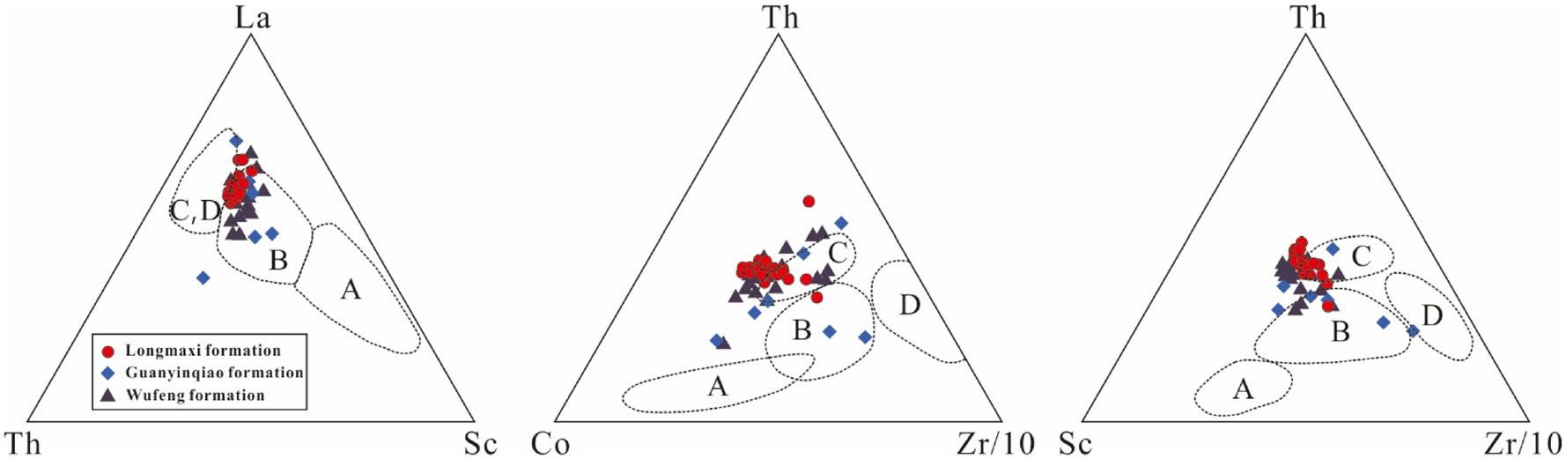
La–Th–Sc, Th–Co–Zr/10 and Th–Sc–Zr/10 plots of the shales and carbonaceous marls from the Wufeng-Longmaxi formations in the Xindi No. 2 well for tectonic discrimination. Dotted lines represent the dominant fields for sedimentary rocks from various tectonic settings (Bhatia and Crook^70^): (**A**) oceanic island arc; (**B**) continental island arc; (**C**) active continental margin; (**D**) passive margin.

Numerous studies have shown that the geochemical characteristics of detrital rocks are significantly controlled by the plate tectonic setting of the source area, therefore, the tectonic setting of the ancient terrains has been widely identified by using major-, trace- and rare earth element-based discrimination diagrams^79–81^. Bhatia and Crook^70^ proposed trace element-based discrimination diagrams to differentiate four tectonic settings: continental island arc, oceanic island arc, active continental margins, and passive margins. As shown in Fig. 10, most of the studied samples fall within or adjacent to the continental island arc and active continental margin domain in diagrams of La–Th–Sc, Th–Co–Zr/10, and Th–Sc–Zr/10.

**Figure 11:**
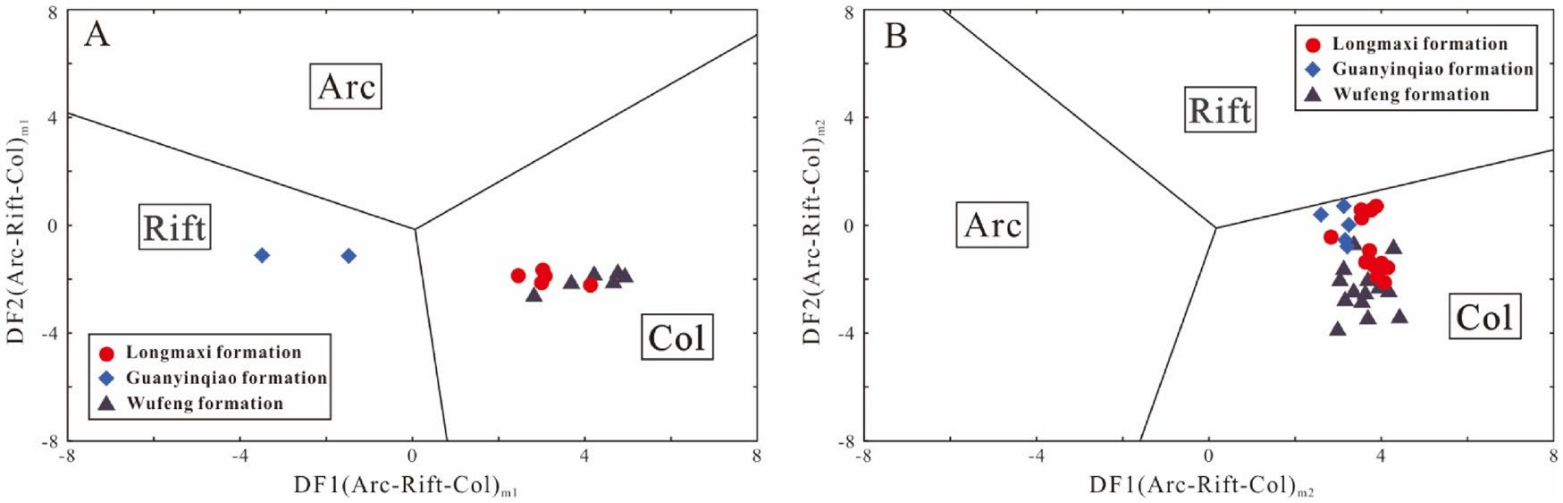
New discriminant function multidimensional diagram proposed by Verma and Armstrong-Altrin^82^. (**A**) High-silica clastic sediments from three tectonic settings (arc, continental rift and collision). (**B**) Low-silica clastic sediments from three tectonic settings (arc, continental rift, and collisional).

To increase the success rate of identifying the tectonic setting, Verma and Armstrong-Altrin^82^ used common oxides (SiO_2_, Al_2_O_3_, Fe_2_O_3_, MgO, CaO, Na_2_O, K_2_O, TiO_2_, P_2_O_5_, and MnO) to develop two multidimensional diagrams based on the log_e_-ratio transformation of major oxides to discriminate arc, continental rift, and collisional settings. All the major oxides must be adjusted to 100% after excluding the loss on ignition (LOI) and regarded as (X)_adj_, where X represents the major oxides. These diagrams can be divided into two types based on the difference in (SiO2)_adj_ values, low-silica type (36%-63%), and high-silica type (63–95%). More details about the calculated equations for these two types of sediments are described in Verma and Armstrong-Altrin^82^. Recently, these diagrams have been successfully applied to discriminate the tectonic setting of older sedimentary basins^83–86^. In the present study, the (SiO_2_)_adj_ contents of the Wufeng-Longmaxi formations vary from 32.47 to 87.15, which can be classified as low-silica samples (n = 41) and high-silica samples (n = 13). For the high-silica diagram (Fig. 11A), eleven high-silica samples from the Wufeng and Longmaxi formations plot in the collision field, with two samples from the Guanyinqiao Formation (XD2P-B31 and XD2P-B29) plotting in the arc field. On the low-silica diagram (Fig. 11B), all forty-one low-silica samples plot in the collision field. All the discriminant-function diagrams above reflect that the sediments of the Wufeng and Longmaxi formations mainly originated from a collisional setting, while the Guanyinqiao Formation may be derived from rift and collisional settings.

### Palaeoweathering indices and palaeoclimate implications

**Figure 12:**
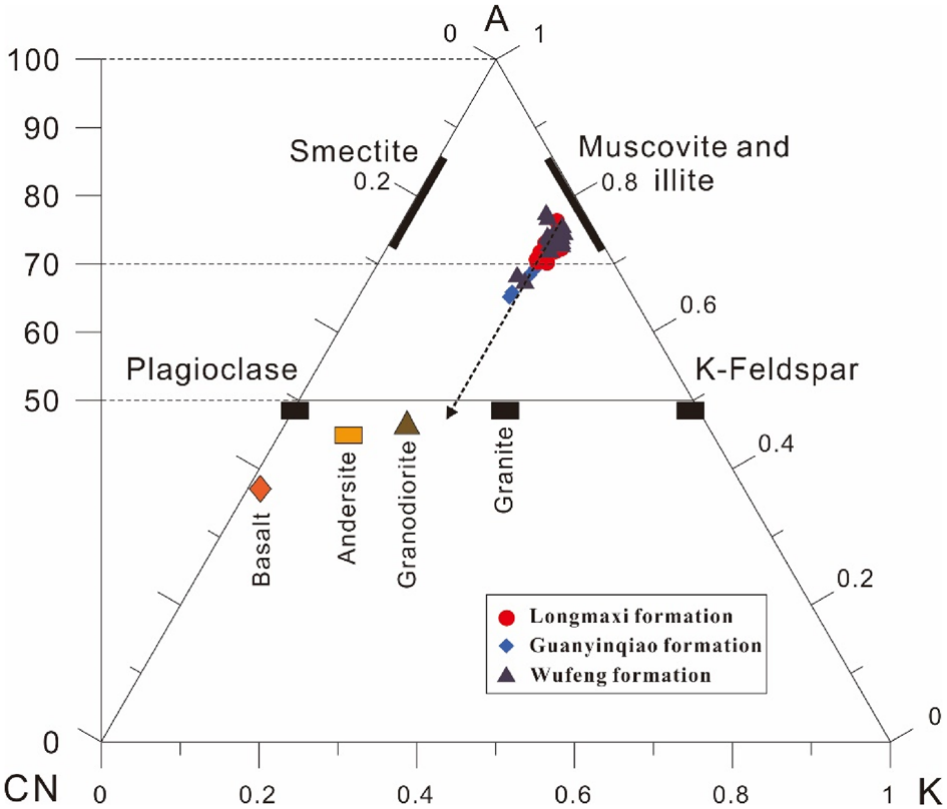
Al_2_O_3_–CaO^*^ + Na_2_O–K_2_O ternary diagram for shales and carbonaceous marls of the Wufeng-Longmaxi Formations in the Xindi No. 2 well (after Nesbitt and Young^91^).

The intensity of chemical weathering is controlled mainly by the source rock composition, during weathering, climatic conditions, and rates of tectonic uplift of the source region (e.g., Refs.^87,88^). During chemical weathering, alkaline metal elements such as Ca, Na, and K are largely removed from source rocks, while the concentrations of Al, Si, and Ba increase in the residue^89,90^. The degree of weathering can be quantified by using mobile and immobile element oxides such as Na_2_O, CaO, K_2_O, and Al_2_O_3_. The ternary diagram of Al_2_O_3_ − (CaO^*^ + Na_2_O) − K_2_O (A–CN–K) (molecular proportions^91^) has been widely used to evaluate the differences in chemical composition related to chemical weathering, which can also be used to analyse the weathering history and palaeoclimate^92–96^. As shown in Fig. 12, the samples of the three formations merged above the plagioclase-K-feldspar join, showed a narrow linear trend and were close to the muscovite and illite fields, which are consistent with the XRD results (Table 1). The weathering trend is parallel to the A-CN borderline and does not show any tilt to the K apex, indicating that the chemical weathering conditions are relatively stable, and excluding the effect of potassium salt metasomatism during diagenesis, which corrects CIA values for further analysis.

Among the different indices of weathering, the CIA is widely used in recent studies to quantify the degree of weathering^97–99^. Young and Nesbitt^100^ proposed that the degree of chemical weathering is related to the climatic conditions in the source area, and is considered a method to study palaeoclimate changes. This discovery also makes CIA indicators widely used to reconstruct palaeoclimate conditions^20,97–99^.

The CIA values of the Wufeng-Longmaxi formation samples were calculated according to the method of McLennan^62^, and the results are listed in Table 2. Across the Ordovician–Silurian boundary, the CIA values of black shales from the Katian stage (from *D. complanatus* through *P. pacificus* biozones) are relatively consistent (Fig. 3), ranging from 67.48 to 73.37 (average of 69.72). In the lower *M.extraordinarius* zone (early Hirnantian), the CIA values, although they apparently decrease to a low, show a more fluctuating pattern in the *M. extraordinarius-M. persculptus* zone, varying between 58.30 and 68.74 (average of 62.62). At the top of the *M. persculptus* zone, they reached 64.66. Farther up, the CIA values return to persistently high values, from 63.47 to 72.85 (average of 67.77) in the Rhuddanian stage (from the *A. ascensus* through *C.vesiculosus* biozones).

In general, a higher CIA value (80–100) indicates tropical and humid climate conditions with strong chemical weathering, a medium CIA value (60–80) indicates moderate chemical weathering accompanied by warm and humid climate conditions, and a lower CIA value (50–60) indicates the palaeoclimate conditions of weak chemical weathering with a cold and arid climate^60,61,97^. The lower CIA value (67.48–73.37) of the lower Wufeng Formation (*D. complanatus*—*M. extraordinarius* biozones) indicates a moderate degree of chemical weathering, which may reflect the warm and humid climatic conditions in the source area. Farther up, the CIA values during the Hirnantian stage (*M. extraordinarius*–*M. persculptus* biozones) first decreased from 69.23 to 61.51, then suddenly increased to 68.74, and then still showed a fluctuating pattern (58.30–64.66). These results indicate that the climatic conditions were mainly cold and dry before deposition, and the sediments experienced weak to moderate weathering, which is consistent with widespread Hirnantian glaciation^101–103^. However, based on the data fluctuations, there may also be short-term pulses of climate warming during this interval. In summary, during the Hirnantian stage, the climate fluctuated rather than was persistently cold and arid.

A similar scenario has been reported in previous studies^97,104,105^, including approximately five glacial-interglacial cycles during the glacial period with an estimated duration of ~ 500^13^ to ~ 1.0-Myr^106^. As shown in Fig. 3, our fluctuating but relatively low CIA values correlate with a pronounced δ^13^C positive excursion, which represents a match with the global δ^13^C Hirnantian excursion (HICE)^107,108^. Following this minimum, the CIA values increased above the Early Rhuddanian (at the bottom of the *A. ascensus* biozone), indicating that chemical weathering gradually increased, but compared to the pre-glaciation sediments (lower part of the Wufeng Formation) the CIA values remained relatively low, which means that the post-glacial climatic conditions remained relatively cool.

### Palaeoredox conditions

Many trace elements, including U, V, Mo, Ni, and Co, show that their oxidation state and solubility change along with the variations in the redox state of the sedimentary environment; thus, they have been widely used to reconstruct palaeoredox conditions of sedimentary rocks and marine sediments^109–113^. Among such elements, uranium (U) and molybdenum (Mo) have been extensively studied due to differences in their geochemical behaviour, and their covariation is considered to be related to specific redox conditions and processes in marine depositional systems^114^. The absorption of authigenic uranium by marine sediments starts under suboxic conditions, while the enrichment of authigenic molybdenum requires the presence of H_2_S (i.e., euxinic conditions)^115–118^. Moreover, aqueous U is completely unaffected through the particulate Mn/Fe-oxyhydroxide shuttle, but aqueous Mo may be enhanced during this process^119,120^. Based on the above differences, the autogenous U–Mo covariance mode can be used to track the redox conditions, which are determined by its EF (see definition in “Major element”). In addition, some redox indices (U/Th, V/Cr, Ni/Co, and V/V + Ni) have been widely used to derive information on the palaeo-oxygen level of depositional environments^121–125^. All four indices were calculated and reported as stratigraphic variations in Fig. 14.

**Figure 13:**
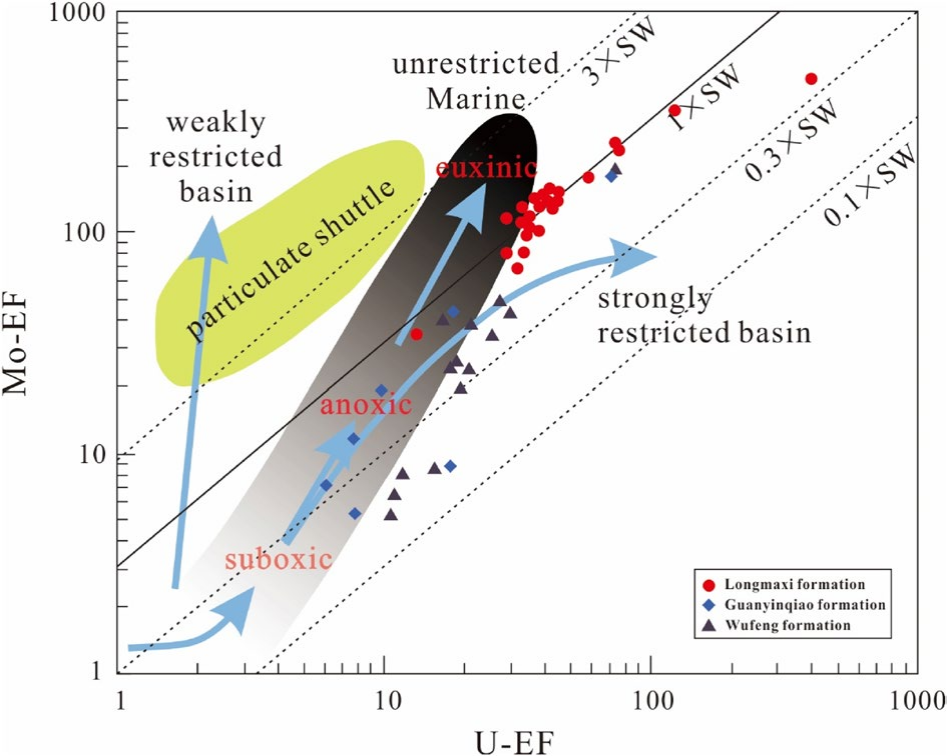
Plot of Mo_EF_ vs. U_EF_ from the studied samples normalized using shale data^56^. The solid line shows the Mo–U molar ratio equal to the seawater value (1 × SW). Dashed lines indicate Mo–U molar ratios equal to fractions of seawater values^110,114^ (3 × SW, 0.3 × SW).

**Figure 14:**
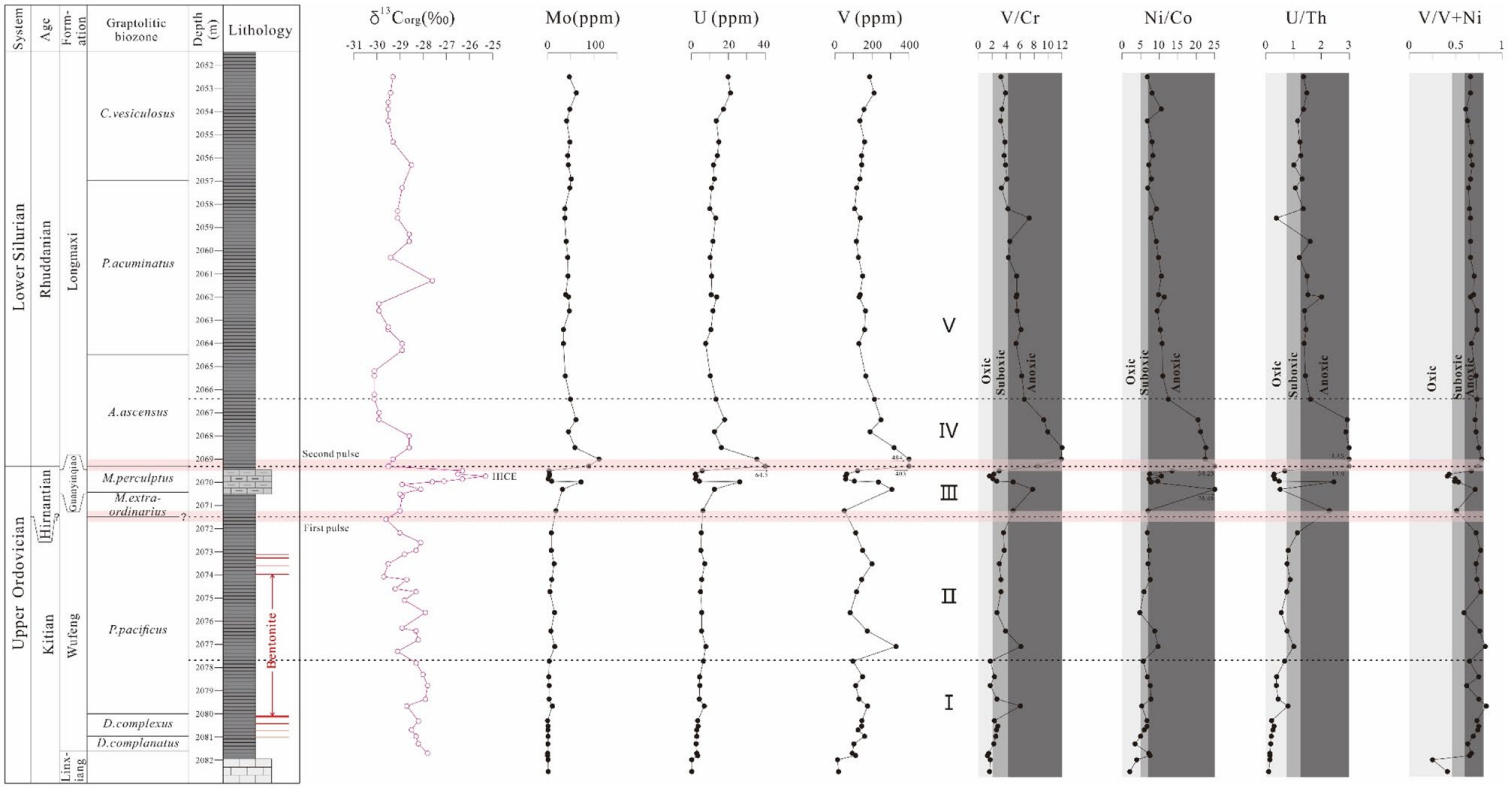
Stratigraphic variations in palaeoredox proxies for the Xindi No. 2 well.

All palaeoredox proxies indicate significant changes in redox conditions during the Late Ordovician to early Silurian, which can be identified as five distinct parts (Figs. 13, 14). For part I, during the accumulation of lower Wufeng formation (*D. complantus*, *D. complexus*, and lower part of *P. Pacificus* graptolite zones), all of the samples exhibit little or no enrichment for Mo and U, with (Mo/U)_auth_ ratios ranging from 0.05 to 1.33 with a median of 0.13, which are mostly between 0.1 and 0.3 times those of seawater (Fig. 13). The U/Th, V/Cr and Ni/Co values range from 0.16 to 0.83 (average of 0.35), 1.36–6.05 (average of 2.48) and 3.48–7.79 (average of 6.24), respectively, pointing to oxic to suboxic bottom water (Fig. 14). The above data suggest fluctuations between oxic and suboxic conditions.

For part II, during the upper Wufeng Formation (upper part of the *P. Pacificus* graptolite zone), most samples display elevated Mo enrichment, ranging from 5.36 to 18.1 ppm, with an average of 11.19 ppm. (Mo/U)_EF_ ratios mostly fluctuate between 0.3 and 1 times seawater, plotting closer to the anoxic end (Fig. 14). The U/Th, V/Cr, and Ni/Co values increase, varying between 0.56 and 2.30 (average of 1.00), 2.64–6.15 (average of 3.84), and 4.85–9.74 (average of 7.25), indicating fluctuations between suboxic and anoxic bottom waters. All of the above data reflect at least intermittent anoxic conditions below the Guanyinqiao Formation.

For part III, during the accumulation of Guanyinqiao Formation (*M. extraordinarius* and *M. persculptus* graptolite zones), most palaeo-redox proxies show first an increase and then a decrease, including Mo and U contents and U/Th, Ni/Co, V/Cr, V/V + Ni ratios. Most samples show low concentrations of Mo (< 10 ppm), but much higher Mo contents (32.60 and 71.80 ppm) occurred at the base of the Guanyinqiao Formation. (Mo/U)_EF_ ratios are generally less than that of seawater (0.3 × SW to 1 SW; Fig. 13), suggesting suboxic bottom water conditions. The U/Th, V/Cr, and V/V + Ni ratios indicate oxic to suboxic conditions (Fig. 14). Although most of the samples point to anoxic conditions, the Ni/Co ratio still shows a trend of increasing first and decreased afterwards. Thus, careful should be taken when applying these criteria to interpret the redox conditions.

For part IV, during the lower Rhuddanian Stage (*A. ascensus*-*C. veiculosus* graptolite zones), most palaeo-redox indices show similar variation patterns to that of part III, which increase first and then decrease. Most samples exhibit high average Mo concentrations of 49.67 ppm (and as large as 111 ppm), with (Mo/U)_EF_ ratios mostly approximately 1 time that of seawater (Fig. 13), suggesting a euxinic condition. Similar conclusions were found in the interpretations of U/Th, V/Cr, Ni/Co, and V/V + Ni ratios (Fig. 14), which indicate persistent strong euxinic bottom water during the deposition of the lower part of the Longmaxi Formation.

For part V, during the middle to upper Rhuddanian Stage (upper part of *A. ascensus–C. veiculosus* graptolite zone), most proxies remain at a steady level compared to those in part IV. The V/Cr, Ni/Co and U/Th ratios range from 3.2 to 7.31 (average of 4.75), 6.84–12.47 (average of 9.2), and 0.38–2.09 (average of 1.35), respectively, reflecting basic anoxic bottom waters. The Mo concentrations maintain a high level (average of 44.1 ppm), with (Mo/U)_EF_ ratios mostly approximately 1 times that of seawater (Fig. 13), revealing euxinic water conditions. The above data suggest that euxinia may have been maintained during the deposition of the lower Longmaxi Formation.

### Relationship of δ^13^C_org_ variations to environmental changes and possible causes of carbon isotope excursions during the O–S interval

Our data that are derived from Xindi No. 2 well as described above clearly show water column redox variations pre-and-post Hirnantian glaciation, which can be summarized as two pulses of deepening degrees of anoxia. A parallel variation in δ^13^C_org_ values is also well documented in the Xindi No. 2 well. The positive δ^13^C_org_ excursion recognized as the HICE was initiated after the first pause of the Hirnantian glaciation from the *M.extraordinarius* zone to the lower *M. persculptus* zone (Figs. 12 and 14), followed by subsequent redox condition variations from increasing anoxia to sudden oxic-suboxic conditions. The δ^13^C_org_ values recorded above the HICE exhibit a − 3.2‰ negative excursion after the second pause of the Hirnantian glaciation from the upper *M. persculptus* zone to the lower *A. ascensus* zone, accompanied by rapid variations from oxic-suboxic to strong euxinic water conditions. These two abrupt shifts display strong covariations between δ^13^C_org_ and redox conditions, indicating a significant change in the relative carbon fluxes between ocean water, organic matter, and sediments.

**Figure 15:**
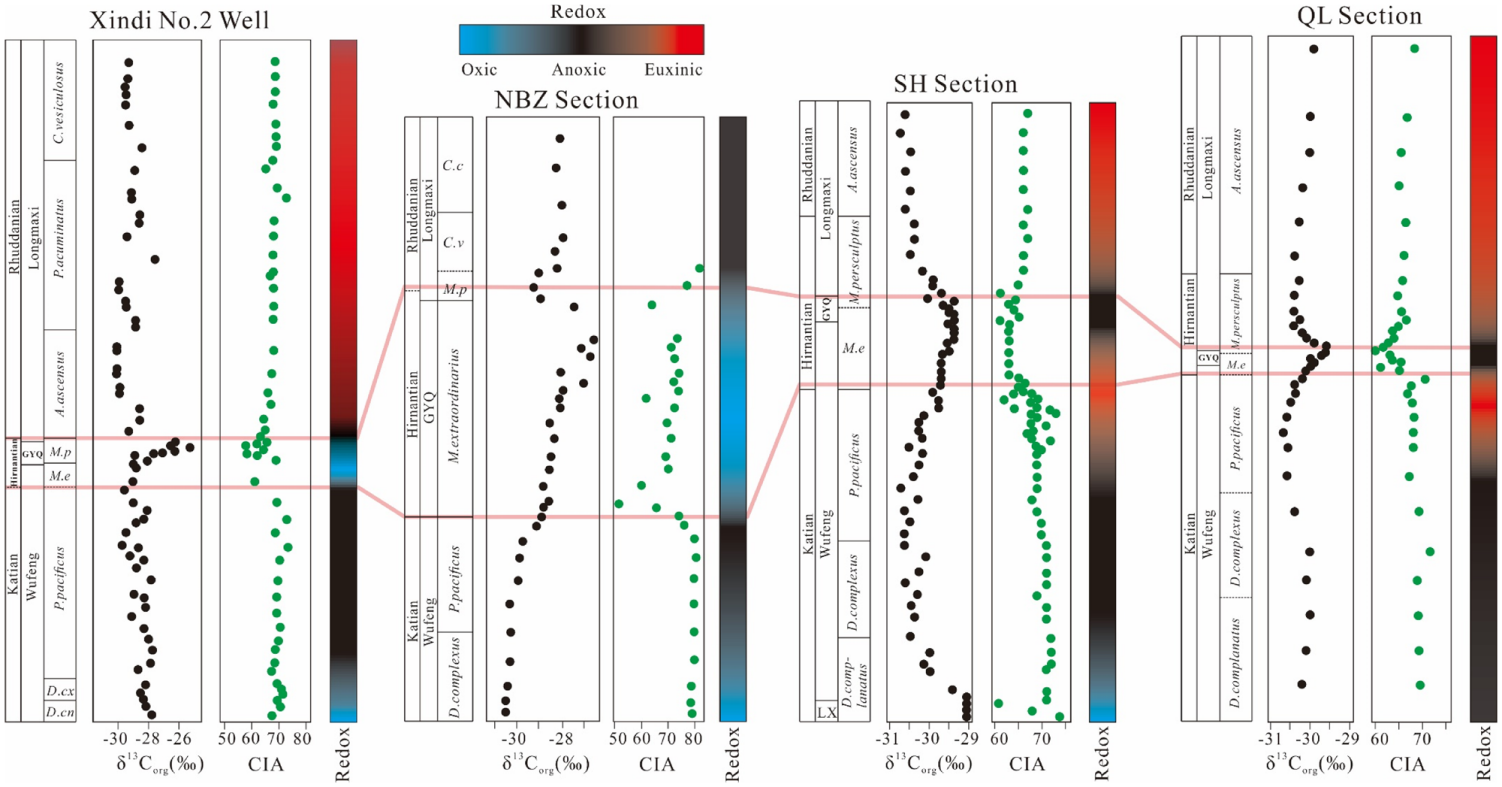
General trends of δ^13^C_org_, climate change, and redox conditions in the Xindi No. 2 well (this study), NBZ^42^, SH, and QL^20^ sections. Graptolite zones: *D. cn.—Dicellograptus complanatus; D. cx.—Dicellograptus complexus; P. pacificus—Paraorthograptus pacificus; M. e.—Metabolograptus extraordinarius; M. p.—Metabolograptus persculptus; A. a.—Akidograptus ascensus.*

In order to explore the spatial variation in the marine carbon isotopes during the Late Ordovician–early Silurian, we compared the lateral and vertical change in δ^13^C_org_ values across proximal to distal areas on the Yangtze Shelf Sea (Fig. 15). Three sections were selected for geochemical analyses^20,42^, along with the Xindi No. 2 well in this study, and each section represents a lateral change from the inner- to mid-shelf. All sections are well established by graptolite biozonation, which can be used as stratigraphic correlation. As is shown in Fig. 15, the positive δ^13^C_org_ excursions characterize the Hirnantian interval across all four sections, but by comparing the positive shift, the records appear to be diachronous. In the shallow-water setting (NBZ) recorded by Yan et al.^40^, the increase in δ^13^C_org_ occurs in the *Metabolograptus extraordinarius* biozone, while this excursion can be observed in the *Metabolograptus persculptus* biozone in a deeper water setting from the inner-shelf (SH section) to the mid-shelf (QL section), as well in the Xindi No. 2 well. The offset of δ^13^C_org_ values vary across all four sections, with a larger offset of ~ 3.7‰ in the Xindi No. 2 well, and of ~ 2.6‰ in the NBZ section, while relatively smaller offset values are present in the SH and QL sections (of ~ 1.2‰ and ~ 1.1‰). We suggest that this δ^13^C_org_ gradient might be explained by the different oceanic environments between shallow- and deep-water settings. SH and QL were under anoxic-euxinic conditions during Hirnantian glaciation according to Fe speciation and Mo concentrations^20^, while the NBZ and Xindi No. 2 well have been established as oxic and oxic-suboxic during the glaciation by Zhou et al.^12^ and by palaeoredox proxies in this study (Fig. 14). These spatial differences in water column redox conditions were probably due to differences in depositional water depths, with shallower waters associated with more oxic conditions.

Positive excursions (both δ^13^C_carb_ and δ^13^C_org_) during the Hirnantian stage have been reported from sections of the Yangtze Platform (e.g. Refs.^3,11,17,30,126,127^) as well as from different regions worldwide(e.g. Refs.^13,14,108,128^). Proposed geological processes that may cause positive δ^13^C excursions include the following: (1) enhanced marine productivity and organic matter burial^13,15,129,130^, (2) increased carbonate-platform weathering^128,131^, (3) dissolved inorganic carbon (DIC) with high δ^13^C^132^, and (4) sea-level eustacy and shoaling of the marine chemocline^38,127,133^. The positive δ^13^C excursion has been interpreted as the result of increased organic carbon burial, which lowered atmospheric *p*CO_2_, leading to a consequent ^13^C enrichment^108,128,134,135^. Due to the fact that photosynthetic carbon fixation of marine phytoplankton favours ^12^C relative to the carbon reservoir, the progressive increase in the burial of organic matter could have led to substantial enrichment of ^13^C in seawater, leading to a positive excursion of δ^13^C (e.g. Refs.^15,128,129,136^. Based on these theories, the HICE is inferred to be indicative of a major carbon burial event during the Late Ordovician. However, this organic carbon burial causal model is yet to be reconciled with the marked lithofacies change, as the shelly limestone of the Guanyinqiao Formation is sandwiched between the pre-glaciation of the Wufeng Formation and the post-glaciation of the Longmaxi Formation black shales. Furthermore, data collected from recent studies have shown low TOC values during the Hinantian interval both on the Yangtze Platform (e.g. Refs.^30,43^) and from global strata (e.g. Refs.^19,137–139^). The carbonate weathering model proposed by Kump et al.^128^ linked the δ^13^C excursion, especially that of δ^13^C_carb_, to the enhanced carbonate platform weathering during glacioeustatic sea-level lowstand. The strong positive δ^13^C_org_ excursion from the Xindi No. 2 well coincident with the glacial Guanyinqiao Formation may reflect increased carbonate weathering during the Hirnantian glaciation. Although there may exhibit short-term pulses of climate warming during the Hirnantian glaciation (Fig. 12), which could accelerate carbonate platform weathering, high-resolution studies are still required to verify this conclusion. Apart from organic carbon burial and carbonate platform weathering, other factors, including dissolved inorganic carbon (δ^13^C_DIC_) and glacio-eustatic sea-level change, cannot be excluded. Dissolved inorganic carbon (DIC) with high δ^13^C values, which were possibly generated by an authigenic carbonate precipitation mechanism, may play an important but poorly understood role in the acknowledgment of the Hirnantian excursion. According to Ahm et al.^19^, glacio-eustatic restriction of shallow epeiric seas may increase both lateral- and vertical gradients in δ^13^C_DIC_ from the shallow shelf to the deep basin. The lateral gradient exhibits higher seawater δ^13^C values in shelf-proximal than in shelf-distal environments due to increased photosynthesis and carbonate weathering, which can be observed in the Canada Arctic and Baltic basins^133,140^ as well in the Yangtze shelf sea^127,141^. This theory may explain such a high δ^13^C_org_ peak (~ − 25.3‰) in the Xindi No. 2 well, as its locality is closer to the shelf-proximal unit compared to previous studies (Fig. 15). Generally, organic matter is produced by photosynthetic organisms in surface water, as a result of the uptake of ^12^C, and is removed as sedimentary organic matter in deep water, causing even higher ^13^C values in proximal settings^142^. A surface- to deep-water gradient has been recorded in the modern Black Sea, which is a typical example of a bottom-sulfurized and stagnant ocean, where the δ^13^C_org_ of photosynthate in surface waters is ~ 4‰ heavier than the δ^13^C_org_ of sedimentary organic matter in sulfidic deep waters^143^. It is worth noting that the Xindi No. 2 well was characterized by the highest δ^13^C_org_ value among all four sections (Fig. 15), which can be explained by two factors. On the one hand, the increasing circulation of organic carbon fluxes, either by photosynthetic activity or by dissolved O_2_, contributed to the preservation of the heavier δ^13^C_org_ under more oxic water relative to those deposited under anoxia water conditions. On the other hand, increasing carbonate weathering exposed by the glacio-eustatic sea-level fall may have further accelerated carbon input to the proximal setting, contributing to the positive δ^13^C_org_ excursion. Thus, our high values of δ^13^C_org_ might be associated with increased photosynthetic activity and carbonate weathering when the sediments of the Xindi No. 2 well were deposited under oxic conditions in a shelf-proximal setting.

### Environmental change in South China during the Hirnantian glaciation

The LOME was marked by two-phase extinction events^144^. The first strike occurred in the *M. extraordinarius–N. ojsuensis* biozone (~ 445 Ma) coinciding with the onset of the Hirnantian glaciation and the positive shift in δ^13^C values of carbonates and organic carbon. The second wave of extinctions occurred in the early *M. persculptus* Zone (~ 444 Ma) coinciding with the subsequent abrupt rise in sea-level marked by a large negative shift in δ^13^C values^1,6,11,20,129,145^. Our δ^13^C_org_ isotopic data provide a good constraint on the timing of the two-phase extinctions, combined with the aforementioned studies, which can also provide the relevant evolution of the temporal changes in climatic and ocean redox conditions (Figs. 8 and 15). From the late Katian (Late Ordovician), the Yangtze Block constantly collided with the Cathaysia Block due to Caledonian movement^146–150^, resulting in northwest–southeast compression that caused the constantly expanding uplifts (i.e., Chengdu, Dianqian, and Jiangnan-Xuefeng uplifts) around the Sichuan basin and a deepening of the inner Yangtze Sea^151–155^. During the deposition of the lower part of the Wufeng Formation, the Yangtze Sea evolved into a semi-isolated shelf sea, and gradually developed suboxic to anoxic bottom waters, which is consistent with all palaeoredox proxies (Mo-auth/U-auth, U/Th, V/Cr, and Ni/Co ratios). The climate of the Upper Yangtze basin during this time was generally warmer and more humid, consistent with CIA values from 67.48 to 73.37 (average of 69.72).

In the early Hirnantian, glaciation started on Gondwana, and its initial phase was related to the beginning of a cool climate. With the continuous formation of ice sheets, the global sea level began to fall, and the climate became colder and drier, which is consistent with the decline in CIA values (from 69.23 to 61.51) at the bottom of the *Normalograptus. extraordinarius* biozone (upper part of the Wufeng Formation). As temperatures decreased and eustatic sea-level dropped, redox conditions varied from anoxic to oxic-suboxic, which is consistent with the geochemical evidence discussed in our previous chapter. The overlying Guanyinqiao Formation thus deposited carbonaceous marls with abundant and diverse shelly fauna. During the early Hirnantian period, the oceanic redox evolution of the entire Yangtze Sea was mainly caused by sea level decline, and the sea level decline itself was the result of rapid global cooling. This result highlights the close relationship between the redox evolution of the Late Ordovician water column and climate change at that time^26,40,156^. These conclusions are supported by the δ^13^C_org_ evidence in the Upper Hirnantian samples (Fig. 15).

In the late Hirnantian stage (*M. persculptus* biozone), glaciation ended, marked by an abrupt eustatic sea level rise. The climate started getting warmer and moister, which is consistent with the increase in the CIA values in the upper part of the Guanyinqiao Formation (from 58.30 to 62.60). As temperatures increased and sea level rose, the water-column redox rapidly returned to euxinia, coincident with the rising ratios (Mo-auth/U-auth, U/Th, V/Cr, Ni/Co, and V/V + Ni ratios) and Mo concentrations. In the early Rudanian stage, sea level rise and widespread euxinia jointly promoted black shale deposition in the Longmaxi Formation.

These data, together with previous research from other areas in the Yangtze Sea, indicate significant climatic fluctuations, from warming to cooling at the end of the late Katian stage, and then back to a warm climate at the end of the late Hirnantian, possibly fluctuate during these two-time intervals. Correspondingly, the marine redox system changed from anoxic to oxygenated, and then suddenly evolved into euxinic bottom waters, consistent with two pulses of the LOME. Climatic fluctuations, coupled with oceanic anoxia, were likely responsible for the gradual end of the Late Ordovician biotic crisis.

## Conclusions

This study aims to reconstruct the depositional history of the Late Ordovician-early Silurian boundary successions in the Xindi No. 2 well deposited on the Yangtze shelf sea and provides new insights into possible drivers of perturbations of the carbon isotope records and possible causes of the LOME. Sediments from the Wufeng-Longmaxi formations were mainly derived from the collisional settings, presumably from active continental margins based on the ternary diagrams and multidimensional diagrams. The provenance of shales and carbonaceous marls from these three formations are mainly from felsic igneous rocks and show weak sediment recycling. The CIA values of the studied samples indicate that the intensity of chemical weathering in the source area was weak to moderate. Lower Wufeng and Longmaxi formations indicate a warm and humid climate during deposition. In contrast, the CIA value suddenly began to decline in the upper part of the Wufeng Formation (Hirnantian stage) and continued to decline until the Guanyinqiao Formation, and data fluctuations during this period indicate that the cold and arid climate was interrupted by brief warm climates. The palaeoredox indices (Mo concentrations and Mo_auth_/U_auth_, U/Th, V/Cr, Ni/Co, and V/V + Ni values) during the Late Ordovician- early Silurian indicate two cycles of water column euxinia. The first cycle occurred during the accumulation of the Wufeng Formation, with bottom waters evolving from oxic-suboxic (part I) to suboxic-anoxic (part II). Most samples show relatively low concentrations of redox-sensitive trace elements during the Guanyinqiao Formation (part III), pointing to oxic-suboxic conditions. The water column transitioned from oxic to euxinic in the late Hirnantian (at the base of the Longmaxi Formation, part IV). Our δ^13^C_org_ data are comparable to previously reported records and exhibit a strong correlation between the HICE, climate change, and redox conditions. We suggest that the variations in δ^13^C values are related to two elements: (1) increased photosynthetic activity under oxic water conditions; (2) increased carbonate weathering exposed by the glacio-eustatic sea-level. In addition, our high values of δ^13^C_org_ compared to the other three previous sections might indicate a more shelf-proximal setting during the deposition of the Xindi No. 2 well. The δ^13^C_org_ isotopic data effectively constrain the timing of the LOME and the evolution of the temporal changes in the climatic and ocean redox conditions, suggesting an apparent stratigraphic coincidence between climate and redox fluctuations and two-phase extinctions, which implies a strong causal relationship. The LOME was systematically driven by the combination of cooler glacial temperatures, glacio-eustatic sea-level fluctuations, and anoxic water conditions that caused the two pulses of extinction in the Yangtze shelf sea.

## Data Availability

All data analysed during this study are included in this published article.

## References

[1] Harper DAT, Hammarlund EU, Rasmussen CMØ (2014). End Ordovician extinctions: A coincidence of causes. Gondwana Res..

[2] Algeo TJ, Marenco PJ, Saltzman MR (2016). Co-evolution of oceans, climate, and the biosphere during the ‘Ordovician Revolution’: A review. Palaeogeogr. Palaeoclimatol. Palaeoecol..

[3] Liu Y, Li C, Algeo TJ, Fan J, Peng PA (2016). Global and regional controls on marine redox changes across the Ordovician-Silurian boundary in South China. Palaeogeogr. Palaeoclimatol. Palaeoecol..

[4] Brenchley P (1984). Late Ordovician extinctions and their relationship to the Gondwana glaciation. Geol. J. Special Issue.

[5] Brenchley PJ, Marshall JD, Underwood CJ (2001). Do all mass extinctions represent an ecological crisis? Evidence from the Late Ordovician. Geol. J..

[6] Rong JY, Chen X, Harper DAT (2002). The latest Ordovician Hirnantia Fauna (Brachiopoda) in time and space. Lethaia.

[7] Chen X, Rong JY, Boucot AJ (2004). Facies patterns and geography of the Yangtze region, South China, through the Ordovician and Silurian transition. Palaeogeogr. Palaeoclimatol. Palaeoecol..

[8] Chen X (2006). The Global Boundary Stratotype Section and Point (GSSP) for the base of the Hirnantian Stage (the uppermost of the Ordovician System). Episodes.

[9] Rasmussen CM, Harper DA (2011). Interrogation of distributional data for the End Ordovician crisis interval: Where did disaster strike?. Geol. J..

[10] Rasmussen CM, Harper DA (2011). Did the amalgamation of continents drive the end Ordovician mass extinctions?. Palaeogeogr. Palaeoclimatol. Palaeoecol..

[11] Fan J, Peng PA, Melchin MJ (2009). Carbon isotopes and event stratigraphy near the Ordovician–Silurian boundary, Yichang, South China. Palaeogeogr. Palaeoclimatol. Palaeoecol..

[12] Zhou L (2015). Changes in marine productivity and redox conditions during the Late Ordovician Hirnantian glaciation. Palaeogeogr. Palaeoclimatol. Palaeoecol..

[13] Brenchley PJ (1994). Bathymetric and isotopic evidence for a short-lived Late Ordovician glaciation in a greenhouse period. Geology.

[14] Finnegan S (2011). The magnitude and duration of Late Ordovician-Early Silurian glaciation. Science.

[15] Brenchley PJ (2003). High-resolution stable isotope stratigraphy of Upper Ordovician sequences: Constraints on the timing of bioevents and environmental changes associated with mass extinction and glaciation. Geol. Soc. Am. Bull..

[16] Fortey RA, Owens RM, Rushton AWA (1989). The palaeogeographic position of the Lake District in the Early Ordovician. Geol. Mag..

[17] Zhang T, Shen Y, Zhan R, Shen S, Chen X (2009). Large perturbations of the carbon and sulfur cycle associated with the Late Ordovician mass extinction in South China. Geology.

[18] Hammarlund EU (2012). A sulfidic driver for the end-Ordovician mass extinction. Earth Planet. Sci. Lett..

[19] Ahm ASC, Bjerrum CJ, Hammarlund EU (2017). Disentangling the record of diagenesis, local redox conditions, and global seawater chemistry during the latest Ordovician glaciation. Earth Planet. Sci. Lett..

[20] Zou C (2018). Ocean euxinia and climate change “double whammy” drove the Late Ordovician mass extinction. Geology.

[21] Derakhshi, M., Ernst, R.E. & Kamo, S.L. Ordovician-Silurian volcanism in northern Iran: Implications for a new Large Igneous Province (LIP) and a robust candidate for the Late Ordovician mass extinction. *Gondwana Res*. (2022).

[22] Jones DS, Martini AM, Fike DA, Kaiho K (2017). A volcanic trigger for the Late Ordovician mass extinction? Mercury data from south China and Laurentia. Geology.

[23] Smolarek-Lach J, Marynowski L, Trela W, Wignall PB (2019). Mercury spikes indicate a volcanic trigger for the late ordovician mass extinction event: An example from a deep shelf of the peri-baltic region. Sci. Rep..

[24] Yang S, Hu W, Wang X, Fan J (2021). Nitrogen isotope evidence for a redox-stratified ocean and eustasy-driven environmental evolution during the Ordovician-Silurian transition. Glob. Planet. Change.

[25] Longman J, Mills BJ, Manners HR, Gernon TM, Palmer MR (2021). Late Ordovician climate change and extinctions driven by elevated volcanic nutrient supply. Nat. Geosci..

[26] Qiu Z (2022). Different controls on the Hg spikes linked the two pulses of the Late Ordovician mass extinction in South China. Sci. Rep..

[27] Bergström SM, Chen X, Gutiérrez-Marco JC, Dronov A (2009). The new chronostratigraphic classification of the Ordovician System and its relations to major regional series and stages and to δ13C chemostratigraphy. Lethaia.

[28] Munnecke A, Calner M, Harper DA, Servais T (2010). Ordovician and Silurian sea–water chemistry, sea level, and climate: A synopsis. Palaeogeogr. Palaeoclimatol. Palaeoecol..

[29] Zhang TG, Shen YN, Zhan RB, Shen SZ, Chen X (2009). Large perturbations of the carbon and sulfur cycle associated with the Late Ordovician mass extinction in South China. Geology.

[30] Yan D, Chen D, Wang Q, Wang J, Wang Z (2009). Carbon and sulfur isotopic anomalies across the Ordovician-Silurian boundary on the Yangtze Platform, South China. Palaeogeogr. Palaeoclimatol. Palaeoecol..

[31] Cramer BD (2011). Revised correlation of Silurian Provincial Series of North America with global and regional chronostratigraphic units and δ^13^C_carb_ chemostratigraphy. Lethaia.

[32] Holmden C (2013). Nd isotope records of late Ordovician sea-level change—Implications for glaciation frequency and global stratigraphic correlation. Palaeogeogr. Palaeoclimatol. Palaeoecol..

[33] Subías I, Villas E, Álvaro JJ (2015). Hirnantian (Late Ordovician) δ^13^C HICE excursion in a North Gondwanan (NE Spain) periglacial setting and its relationship to glacioeustatic fluctuations. Chem. Erde-Geochem..

[34] Harper DA, Hints L (2016). Hirnantian (Late Ordovician) brachiopod faunas across Baltoscandia: A global and regional context. Palaeogeogr. Palaeoclimatol. Palaeoecol..

[35] Li Y, Schieber J, Fan T, Li Z, Zhang J (2017). Regional depositional changes and their controls on carbon and sulfur cycling across the Ordovician-Silurian boundary, northwestern Guizhou, South China. Palaeogeogr. Palaeoclimatol. Palaeoecol..

[36] Li Y (2021). Carbon and sulfur isotope variations through the Upper Ordovician and Lower Silurian of South China linked to volcanism. Palaeogeogr. Palaeoclimatol. Palaeoecol..

[37] Bergström SM, Schmitz B (2006). First record of the Hirnantian (Upper Ordovician) δ^13^C excursion in the North American Midcontinent and its regional implications. Geol. Mag..

[38] LaPorte DF (2009). Local and global perspectives on carbon and nitrogen cycling during the Hirnantian glaciation. Palaeogeogr. Palaeoclimatol. Palaeoecol..

[39] Zhang T, Shen Y, Algeo TJ (2010). High-resolution carbon isotopic records from the Ordovician of South China: Links to climatic cooling and the Great Ordovician Biodiversification Event (GOBE). Palaeogeogr. Palaeoclimatol. Palaeoecol..

[40] Yan D, Chen D, Wang Q, Wang J (2012). Predominance of stratified anoxic Yangtze Sea interrupted by short-term oxygenation during the Ordo-Silurian transition. Chem. Geol..

[41] Melchin MJ, Mitchell CE, Holmden C, Štorch P (2013). Environmental changes in the Late Ordovician–early Silurian: Review and new insights from black shales and nitrogen isotopes. Bulletin.

[42] Luo G (2016). Perturbation of the marine nitrogen cycle during the Late Ordovician glaciation and mass extinction. Palaeogeogr. Palaeoclimatol. Palaeoecol..

[43] Li Y, Zhang T, Ellis GS, Shao D (2017). Depositional environment and organic matter accumulation of Upper Ordovician-Lower Silurian marine shale in the Upper Yangtze Platform, South China. Palaeogeogr. Palaeoclimatol. Palaeoecol..

[44] Metcalfe I (1994). Late Palaeozoic and Mesozoic palaeogeography of eastern Pangea and Tethys. Can. Soc. Pet. Geol. Mem..

[45] Torsvik TH, Cocks LRM (2013). Gondwana from top to base in space and time. Gondwana Res..

[46] Chen HD, Huang FX, Xu SL, Zhao LQ, Hua XS (2009). Distribution rule and main controlling factors of the carbonate rock reservoirs in the Middle and Upper Yangtze Region (in Chinese with English abstract). J. Mineral. Petrol..

[47] Mou CL, Xu XS (2010). Sedimentary evolution and petroleum geology in south China during the early palaeozoic (in Chinese with English abstract). Sediment. Geol. Tethyan Geol..

[48] Mou CL, Zhou KK, Liang W, Ge XY (2011). Early paleozoic sedimentary environment of hydrocarbon source rocks in the middle-upper Yangtze region and petroleum and gas exploration (in Chinese with English abstract). Acta Geol. Sin..

[49] Mou CL (2016). Lithofa Palaeogeogra Atlas of China (in Chinese with English Abstract).

[50] Men X, Mou C, Ge X, Wang Y (2020). Geochemical characteristics of siliceous rocks of Wufeng Formation in the Late Ordovician, South China: Assessing provenance, depositional environment, and formation model. Geol. J..

[51] Mu E (1981). Paleogeographic maps of the Late Ordovician in the Central China region and their explanation. J. Stratigr..

[52] Wang LS (2009). Geochemical evidence of shale gas existed in the Lower Paleozoic Sichuan basin (in Chinese with English abstract). Nat. Gas. Ind..

[53] Zhao JH (2016). The genesis of quartz in Wufeng-Longmaxi gas shales, Sichuan Basin (in Chinese with English abstract). Nat. Gas. Geosci..

[54] Zhang, D *et al*. Graptolite biozonation of the wufeng and lungmachi formations and its environmental implication from the xindi 2 borehole in Yongshan–Daguan area, NE Yunnan (in Chinese with English abstract). *J. Earth Sci*. 1–20 (2019).

[55] Ross DJ, Bustin RM (2009). Investigating the use of sedimentary geochemical proxies for paleoenvironment interpretation of thermally mature organic-rich strata: Examples from the Devonian-Mississippian shales, Western Canadian Sedimentary Basin. Chem. Geol..

[56] Tribovillard N, Algeo TJ, Lyons T, Riboulleau A (2006). Trace-metals as paleoredox and paleoproductivity proxies: An update. Chem. Geol..

[57] Ross DJ, Bustin RM (2008). Characterizing the shale gas resource potential of Devonian-Mississippian strata in the Western Canada sedimentary basin: Application of an integrated formation evaluation. AAPG Bull..

[58] Yarincik KM, Murray RW, Peterson LC (2000). Climatically sensitive eolian and hemipelagic deposition in the Cariaco Basin, Venezuela, over the past 578,000 years: Results from Al/Ti and K/Al. Paleoceanography.

[59] Nesbitt HW, Young GM (1982). Early Proterozoic climates and plate motions inferred from major element chemistry of lutites. Nature.

[60] Fedo CM, Young GM, Nesbitt GM (1997). Paleoclimatic control on the composition of the paleoproterozoic serpent formation, huronian supergroup, Canada: A greenhouse to icehouse transition. Precambrian Res..

[61] Nesbitt HW, Young GM (1989). Formation and diagenesis of weathering profiles. J. Geol..

[62] McLennan, S. M., Hemming, S., McDaniel, D. K. & Hanson, G. N. Geochemical approaches to sedimentation, provenance, and tectonics. *Special Papers-Geol. Soc. Am*. 21–21 (1993).

[63] McLennan SM (1989). Rare earth elements in sedimentary rocks: influence of provenance and sedimentary processes. Geochem. Mineral. Rare Earth Elements Rev. Mineral..

[64] Taylor SR, McLennan SM (1985). The Continental Crust: Its Composition and Evolution.

[65] Armstrong-Altrin JS (2015). Provenance and depositional history of continental slope sediments in the Southwestern Gulf of Mexico unraveled by geochemical analysis. Cont. Shelf Res..

[66] Madhavaraju, J. Geochemistry of late Cretaceous sedimentary rocks of the Cauvery Basin, South India: Constraints on paleo-weathering, provenance, and end Cretaceous environments. *Chemostratigraphy*. 185–214 (2015).

[67] Floyd PA, Leveridge BE (1987). Tectonic environments of the Devonian Gramscatho basin, south Cornwall: Framework mode and geochemical evidence from turbidite sandstones. J. Geol. Soc..

[68] Roser BP, Korsch RJ (1988). Provenance signatures of sandstoneemudstone suites determined using discriminant function analysis of major element data. Chem. Geol..

[69] Hayashi KI, Fujisawa H, Holland HD, Ohmoto H (1997). Geochemistry of ca. 1.9 Ga sedimentary rocks from northeastern Labrador. Canada. Geochim. Cosmochim. Acta.

[70] Bhatia MR, Crook KAW (1986). Trace element characteristics of graywackes and tectonic setting discrimination of sedimentary basins. Contrib. Mineral. Petrol..

[71] Armstrong-Altrin JS (2009). Provenance of sands from Cazones, Acapulco, and Bahía Kino beaches, Mexico. Rev. Mex. Cienc. Geol..

[72] Armstrong-Altrin JS (2012). Geochemistry of beach sands along the Western Gulf of Mexico, Mexico: implication for provenance. Chem. Erde.

[73] Fu XG, Wang J, Zeng YH, Tan FW, He JL (2011). Geochemistry and origin of rare earth elements (REEs) in the Shengli River oil shale, northern Tibet, China. Chem. Erde.

[74] Bai YY (2015). Rare earth and major element geochemistry of Eocene fine-grained sediments in oil shale- and coal-bearing layers of the Meihe Basin, Northeast China. J. Asian Earth Sci..

[75] Roddaz M (2006). Controls on weathering and provenance in the Amazonian foreland basin: Insights from major and trace element geochemistry of Neogene Amazonian sediments. Chem. Geol..

[76] Moradi AV, Sarı A, Akkaya P (2016). Geochemistry of the Miocene oil shale (Hançili Formation) in the Çankırı-Çorum Basin, Central Turkey: Implications for Paleoclimate conditions, source-area weathering, provenance and tectonic setting. Sediment. Geol..

[77] Bhatia MR (1983). Plate tectonics and geochemical composition of sandstones. J. Geol..

[78] Piovano EL, Ross GR, Guevara SR, Arribére MA, Depetris PJ (1999). Geochemical tracers of source rocks in a Cretaceous to Quaternary sedimentary sequence (Eastern Sierras Pampeanas, Argentina). J. S. Am. Earth Sci..

[79] Purevjav N, Roser B (2012). Geochemistry of Devonian-Carboniferous clastic Sediments of the Tsetserleg terrane, Hangay Basin, central Mongolia: Provenance, source weathering, and tectonic setting. Isl. Arc.

[80] Tao HF, Sun S, Wang ZQ, Yang XF, Jiang L (2014). Petrography and geochemistry of lower carboniferous greywacke and mudstones in Northeast Junggar, China: Implications for provenance, source weathering, and tectonic setting. J. Asian Earth Sci..

[81] Zaid SM (2015). Geochemistry of sandstones from the Pliocene Gabir Formation, north Marsa Alam, Red Sea, Egypt: Implication for provenance, weathering and tectonic setting. J. Afr. Earth Sc..

[82] Verma SP, Armstrong-Altrin JS (2013). New multi-dimensional diagrams for tectonic discrimination of siliciclastic sediments and their application to Precambrian basins. Chem. Geol..

[83] Armstrong-Altrin JS, Natalhy-Pineda O (2014). Microtextures of detrital sand grains from the Tecolutla, Nautla, and Veracruz beaches, western Gulf of Mexico, Mexico: Implications for depositional environment and palaeoclimate. Arab. J. Geosci..

[84] Armstrong-Altrin JS, Nagarajan R, Balaram V, Natalhy-Pineda O (2015). Petrography and geochemistry of sands from the Chachalacas and Veracruz beach areas, western Gulf of Mexico, Mexico: Constraints on provenance and tectonic setting. J. S. Am. Earth Sci..

[85] Verma SP, Armstrong-Altrin JS (2016). Geochemical discrimination of siliciclastic sediments from active and passive margin settings. Sediment. Geol..

[86] Tawfik, H.A. *et al*. Petrography and geochemistry of the siliciclastic Araba Formation (Cambrian), east Sinai, Egypt: Implications for provenance, tectonic setting and source weathering. *Geol. Mag*. (2016).

[87] Akarish AI, El-Gohary AM (2008). Petrography and geochemistry of lower Paleozoic sandstones, East Sinai, Egypt: Implications for provenance and tectonic setting. J. Afr. Earth Sc..

[88] Moosavirad SM, Janardhana MR, Sethumadhav MS, Moghadam MR, Shankara M (2011). Geochemistry of lower Jurassic shales of the Shemshak Formation, Kerman Province, Central Iran: Provenance, source weathering and tectonic setting. Chem. Erde. Geochem..

[89] Fedo CM, Eriksson KA, Krogstad EJ (1996). Geochemistry of shales from the Archean (3.0 Ga) Buhwa Greenstone Belt, Zimbabwe:implications for provenance and sourcearea weathering. Geochem. Cosmochim. Acta..

[90] Nath BN, Kunzendorf H, Pluger WL (2000). Influence of provenance, weathering, and sedimentary processes on the elemental ratios of the fine-grained fraction of the bedload sediments from the Vembanad Lake and the adjoining continental shelf, southwest coast of India. J. Sediment. Res..

[91] Nesbitt HW, Young GM (1984). Prediction of some weathering trends of plutonic and volcanic rocks based on thermodynamic and kinetic considerations. Geochem. Cosmochim. Acta.

[92] Fedo CM, Nesbitt HW, Young GM (1995). Unraveling the effects of K metasomatism in sedimentary rocks and paleosols with implications for palaeoweathering conditions and provenance. Geology.

[93] Panahi A, Young GM, Rainbird RH (2000). Behavior of major and trace elements (including REE) during Paleoproterozoic pedogenesis and diagenetic alteration of an Archean granite near Ville Marie, Quebec, Canada. Geochim. Cosmochim. Acta.

[94] Fadipe OA, Carey PF, Akinlua A, Adekola SA (2011). Provenance, diagenesis and reservoir quality of the lower cretaceous sandstone of the Orange Basin, South Africa. S. Afr. J. Geol..

[95] Ghosh S, Sarkar S, Ghosh P (2012). Petrography and major element geochemistry of the Permo-Triassic sandstones, central India: Implications for provenance in an intracratonic pull-apart basin. J. Asian Earth Sci..

[96] Deepthi K (2013). Geochemical characteristics and depositional environment of Kalpakkam, Southeast coast of India. Environ. Earth. Sci..

[97] Yan DT, Chen DZ, Wang QC, Wang JG (2010). Large-scale climate fluctuations in the latest Ordovician on the Yangtze block, south China. Geology.

[98] Wang Z (2017). Organic material accumulation of Carnian mudstones in the North Qiangtang Depression, eastern Tethys: Controlled by the paleoclimate, paleoenvironment, and provenance. Mar. Pet. Geol..

[99] Ge X (2019). The geochemistry of the sedimentary rocks from the Huadi No. 1 well in the Wufeng-Longmaxi formations (Upper Ordovician-Lower Silurian), South China, with implications for paleoweathering, provenance, tectonic setting and paleoclimate. Marine Petroleum Geol..

[100] Young GM, Wayne Nesbitt H (1999). Paleoclimatology and provenance of the glaciogenic Gowganda Formation (Paleoproterozoic), Ontario, Canada: A chemostratigraphic approach. Geol. Soc. Am. Bull..

[101] Bergström SM, Huff WD, Kolata DR, Bauert HB (1995). Nomenclature, stratigraphy chemical fingerprinting, and real distribution of some middle Ordovician bentonites in Baltoscandia. GFF.

[102] Astini RA, Collo G, Martina F (2007). Ordovician K-bentonites in the upper-plate active margin of Western Gondwana, (Famatina Ranges): Stratigraphic and paleogeographic significance. Gondwana Res..

[103] Yan DT, Chen DZ, Wang QC, Wang JG (2009). Geochemical changes across the ordovician–silurian transition on the Yangtze platform, south China. Sci. China Earth Sci..

[104] Ghienne JF, Le Heron DP, Moreau J, Denis M, Deynoux M (2007). The Late Ordovician glacial sedimentary system of the North Gondwana platform. Glacial Sedimentary Process. Products..

[105] Turner BR, Armstrong HA, Wilson CR, Makhlouf IM (2012). High frequency eustatic sea-level changes during the Middle to early Late Ordovician of southern Jordan: Indirect evidence for a Darriwilian Ice Age in Gondwana. Sediment. Geol..

[106] Holmden C, Panchuk K, Finney SC (2012). Tightly coupled records of Ca and C isotope changes during the Hirnantian glaciation event in an epeiric sea setting. Geochimica et Cosmochim Acta.

[107] Underwood CJ, Crowley SF, Marshall JD, Brenchley PJ (1997). High-resolution carbon isotope stratigraphy of the basal Silurian stratotype (Dob's Linn, Scotland) and its global correlation. J. Geol. Soc. Lond..

[108] Saltzman MR, Young SA (2005). Long-lived glaciation in the Late Ordovician? Isotopic and sequence-stratigraphic evidence from western Laurentia. Geology.

[109] Barnes CE, Cochran JK (1990). Uranium removal in oceanic sediments and the oceanic U balance. Earth Planet. Sci. Lett..

[110] Algeo TJ, Tribovillard N (2009). Environmental analysis of paleoceanographic systems based on molybdenum-uranium covariation. Chem. Geol..

[111] Partin CA (2013). Large-scale fluctuations in Precambrian atmospheric and oceanic oxygen levels from the record of U in shales. Earth Planetary Sci. Lett..

[112] Trela W, Podhalańska T, Smolarek J, Marynowski L (2016). Llandovery green/grey and black mudrock facies of the northern Holy Cross Mountains (Poland) and their relation to early Silurian sea-level changes and benthic oxygen level. Sed. Geol..

[113] Smolarek J, Marynowski L, Trela W, Kujawski P, Simoneit BR (2017). Redox conditions and marine microbial community changes during the end-Ordovician mass extinction event. Global Planet. Change.

[114] Tribovillard N, Algeo TJ, Baudin F, Riboulleau A (2012). Analysis of marine environmental conditions based on molybdenum–uranium covariation—Applications to Mesozoic paleoceanography. Chem. Geol..

[115] Helz GR (1996). Mechanisms of molybdenum removal from the sea and its concentration in black shales: EXAFS evidence. Geochimica et Cosmochim Acta.

[116] Helz GR, Bura-Nakić E, Mikac N, Ciglenečki I (2011). New model for molybdenum behavior in euxinic waters. Chem. Geol..

[117] Morford JL, Russell AD, Emerson S (2001). Trace metal evidence for changes in the redox environment associated with the transition from terrigenous clay to diatomaceous sediment, Saanich Inlet, BC. Mar. Geol..

[118] Tribovillard N, Riboulleau A, Lyons T, Baudin F (2004). Enhanced trapping of molybdenum by sulfurized organicmatter ofmarine origin as recorded by various Mesozoic formations. Chem. Geol..

[119] Tossell JA (2005). Calculating the partitioning of the isotopes of Mo between oxidic and sulfidic species in aqueous solution. Geochimica Cosmochimica Acta.

[120] Dellwig O (2010). A new particulate Mn–Fe–P-shuttle at the redoxcline of anoxic basins. Geochim. Cosmochim. Acta.

[121] Algeo TJ, Maynard JB (2004). Trace-element behavior and redox facies in core shales of Upper Pennsylvanian Kansas-type cyclothems. Chem. Geol..

[122] Calvert SE, Pedersen TF (1993). Geochemistry of Recent oxic and anoxic marine sediments: Implications for the geological record. Mar. Geol..

[123] Hatch JR, Leventhal JS (1992). Relationship between inferred redox potential of the depositional environment and geochemistry of the Upper Pennsylvanian (Missourian) Stark Shale Member of the Dennis Limestone, Wabaunsee County, Kansas, USA. Chem. Geol..

[124] Jones B, Manning DA (1994). Comparison of geochemical indices used for the interpretation of palaeoredox conditions in ancient mudstones. Chem. Geol..

[125] Wignall PB, Myers KJ (1988). Interpreting benthic oxygen levels in mudrocks: A new approach. Geology.

[126] Xi Z (2021). Geochemical characteristics of organic carbon and pyrite sulfur in Ordovician-Silurian transition shales in the Yangtze Platform, South China: Implications for the depositional environment. Palaeogeogr. Palaeoclimatol. Palaeoecol..

[127] Yang X (2021). Spatiotemporal variations of sedimentary carbon and nitrogen isotopic compositions in the Yangtze Shelf Sea across the Ordovician-Silurian boundary. Palaeogeogr. Palaeoclimatol. Palaeoecol..

[128] Kump LR, Arthur MA, Patzkowsky ME, Gibbs MT, Pinkus DS, Sheehan PM (1999). A weathering hypothesis for glaciation at high atmospheric pCO_2_ during the Late Ordovician. Palaeogeogr. Palaeoclimatol. Palaeoecol..

[129] Marshall JD (1997). Global carbon isotopic events associated with mass extinction and glaciation in the late Ordovician. Palaeogeogr. Palaeoclimatol. Palaeoecol..

[130] Chen Y, Cai C, Qiu Z, Lin W (2021). Evolution of nitrogen cycling and primary productivity in the tropics during the Late Ordovician mass extinction. Chem. Geol..

[131] Jones DS (2011). Terminal Ordovician carbon isotope stratigraphy and glacioeustatic sea-level change across Anticosti Island (Québec, Canada). Bulletin.

[132] Jones DS, Creel RC, Rios BA (2016). Carbon isotope stratigraphy and correlation of depositional sequences in the Upper Ordovician Ely Springs Dolostone, eastern Great Basin, USA. Palaeogeogr. Palaeoclimatol. Palaeoecol..

[133] Melchin MJ, Holmden C (2006). Carbon isotope chemostratigraphy in Arctic Canada: Sea-level forcing of carbonate platform weathering and implications for Hirnantian global correlation. Palaeogeogr. Palaeoclimatol. Palaeoecol..

[134] Arthur MA, Dean WE, Pratt LM (1988). Geochemical and climatic effects of increased marine organic carbon burial at the Cenomanian/Turonian boundary. Nature.

[135] Patzkowsky ME, Slupik LM, Arthur MA, Pancost RD, Freeman KH (1997). Late Middle Ordovician environmental change and extinction: Harbinger of the Late Ordovician or continuation of Cambrian patterns?. Geology.

[136] Marshall JD, Middleton PD (1990). Changes in marine isotopic composition and the late Ordovician glaciation. J. Geol. Soc..

[137] Melchin MJ, Mitchell CE, Barnes CR, Williams SH (1991). Late Ordovician extinction in the Graptoloidea. Adv. Ordovician Geol..

[138] Hallam A, Wignall PB (1999). Mass extinctions and sea-level changes. Earth Sci. Rev..

[139] Melchin MJ, Holmden C (2006). Carbon isotope chemostratigraphy in Arctic Canada: Sea-level forcing of carbonate platform weathering and implications for Hirnantian global correlation. Palaeogeogr. Palaeoclimatol. Palaeoecol..

[140] LaPorte DF (2009). Local and global perspectives on carbon and nitrogen cycling during the Hirnantian glaciation. Palaeogeogr. Palaeoclimatol. Palaeoecol..

[141] Yang X (2021). "Spatiotemporal variations of sedimentary carbon and nitrogen isotopic compositions in the Yangtze Shelf Sea across the Ordovician-Silurian boundary. Palaeogeogr. Palaeoclimatol. Palaeoecol..

[142] Mackensen A, Schmiedl G (2019). Stable carbon isotopes in paleoceanography: Atmosphere, oceans, and sediments. Earth Sci. Rev..

[143] Fry B (1991). Stable isotope studies of the carbon, nitrogen and sulfur cycles in the Black Sea and the Cariaco Trench. Deep Sea Research Part A. Oceanogr. Res. Papers..

[144] Brenchley PJ, Carden GAF, Marshall JD (1995). Environmental changes associated with the “first strike” of the Late Ordovician mass extinction. Mod. Geol..

[145] Rong, J.Y. Distribution of the Hirnantia fauna and its meaning. in *Aspects of the Ordovician System. Palaeontological Contributions from the University of Oslo* (Bruton, D.L. Ed.), Vol. **295**, 101–112 (1984).

[146] Li ZM, Chen JQ, Gong SY, Su WB (1997). Migrate of carbonate platform margin and sea level changes of ordovician in the northwestern hunan, China (in Chinese with English abstract). Earth Sci..

[147] Liu BJ, Xu XS, Pan XN, Huang HQ, Xu Q (1993). The Editorial Broad for Collected Works of Lithofacies and the Crustal Evolution of South China Palaeocontinents (in Chinese with English Abstract).

[148] Cocks LRM, Torsvik TH (2005). Baltica from the late Precambrian to mid-Palaeozoic times: The gain and loss of a terrane's identity. Earth Sci. Rev..

[149] Cocks LRM, Torsvik TH (2011). The Palaeozoic geography of Laurentia: A stable craton with mobile margins. Earth Sci. Rev..

[150] Su WB (2009). K-bentonite, black-shale and flysch successions at the Ordovician-Silurian transition, South China: Possible sedimentary responses to the accretion of Cathaysia to the Yangtze Block and its implications for the evolution of Gondwana. Gondwana Res..

[151] Charvet J (2010). Structural development of the Lower Paleozoic belt of South China: Genesis of an intracontinental orogen. J. Asian Earth Sci..

[152] Liu W, Xu XS, Feng XT, Sun YY (2010). Radiolarian silicalites and palaeoenvironmental reconstruction for the upper Ordovician Wufeng formation in the middleupper Yangtze area (in Chinese with English abstract). Sediment. Geol. Tethyan Geol..

[153] Liu W (2012). Lithofacies palaeogeography of the late ordovician hirnantian in the middle-upper Yangtze region of China (in Chinese with English abstract). J. Chengdu Univ. Tech..

[154] Chen X (2012). Onset of the Kwangsian Orogeny as evidenced by biofacies and lithofacies. Sci. China D Earth Sci..

[155] Qiu Z, Zou CN (2020). Unconventional petroleum sedimentology: Connotation and prospect. Acta Sedimentol. Sin..

[156] Qiu Z (2021). Controlling factors on organic matter accumulation of marine shale across the Ordovician-Silurian transition in South China: Constraints from trace-element geochemistry. J. Earth Sci..

